# A Toolkit for Profiling the Immune Landscape of Pediatric Central Nervous System Malignancies

**DOI:** 10.3389/fimmu.2022.864423

**Published:** 2022-04-07

**Authors:** Jacob S. Rozowsky, Joyce I. Meesters-Ensing, Julie A. S. Lammers, Muriël L. Belle, Stefan Nierkens, Mariëtte E. G. Kranendonk, Lennart A. Kester, Friso G. Calkoen, Jasper van der Lugt

**Affiliations:** ^1^ Princess Máxima Center for Pediatric Oncology, Utrecht, Netherlands; ^2^ Center for Translational Immunology, University Medical Center Utrecht, Utrecht, Netherlands

**Keywords:** tumor immune microenvironment, immunotherapy, immune monitoring, central nervous system malignancy, immunohistochemistry, flow cytometry, transcriptomics

## Abstract

The prognosis of pediatric central nervous system (CNS) malignancies remains dismal due to limited treatment options, resulting in high mortality rates and long-term morbidities. Immunotherapies, including checkpoint inhibition, cancer vaccines, engineered T cell therapies, and oncolytic viruses, have promising results in some hematological and solid malignancies, and are being investigated in clinical trials for various high-grade CNS malignancies. However, the role of the tumor immune microenvironment (TIME) in CNS malignancies is mostly unknown for pediatric cases. In order to successfully implement immunotherapies and to eventually predict which patients would benefit from such treatments, in-depth characterization of the TIME at diagnosis and throughout treatment is essential. In this review, we provide an overview of techniques for immune profiling of CNS malignancies, and detail how they can be utilized for different tissue types and studies. These techniques include immunohistochemistry and flow cytometry for quantifying and phenotyping the infiltrating immune cells, bulk and single-cell transcriptomics for describing the implicated immunological pathways, as well as functional assays. Finally, we aim to describe the potential benefits of evaluating other compartments of the immune system implicated by cancer therapies, such as cerebrospinal fluid and blood, and how such liquid biopsies are informative when designing immune monitoring studies. Understanding and uniformly evaluating the TIME and immune landscape of pediatric CNS malignancies will be essential to eventually integrate immunotherapy into clinical practice.

## Introduction

Central nervous system (CNS) malignancies are the leading cause of cancer-related death in children. Extensive molecular profiling has resulted in a better understanding and further subclassification of many pediatric CNS tumors (1). So far, these significant advances are not reflected in clinical benefit for the patients, and the prognosis remains dismal for most of the subtypes. Five-year survival rates for children range from 2% for diffuse midline glioma, 20% for glioblastoma, to 75% for medulloblastoma and ependymoma (2, 3).

Current treatment of children with CNS malignancies is facing a lot of challenges. The diffuse infiltration of some high-grade malignancies into critical neural circuits only permits partial resection or biopsy (4, 5). Depending on diagnosis and age of the patient, either craniospinal or local radiotherapy is added to optimize survival and reduce the risk of (local) recurrence (6). However, radiation can predispose patients to hearing, endocrine, and neuro-cognitive dysfunction (7). Despite the fact that the blood–brain barrier restricts the bioavailability of most of the currently available anti-neoplastic medication, chemotherapy can improve survival. This holds true for the embryonal tumors in particular, though tumors from glial origin tend to be more intrinsically resistant (8). Alternative therapeutic approaches such as molecular-targeted therapies, in particular MEK, BRAF, and tyrosine kinase inhibitors, have manifested improved outcomes in pediatric low-grade and infantile hemispheric gliomas (9). In summary, these highly aggressive and heterogeneous malignancies require an arsenal of agents to achieve meaningful clinical improvement albeit at the cost of significant long-term morbidity. It is clear that conventional treatments fall short, stressing the urge to explore alternative therapeutic modalities.

The field of immunotherapy is a promising area to investigate in the field of pediatric CNS malignancies given its previous success in other solid tumors and superior safety-profile (10–14). Immunomodulating approaches that are currently under investigation comprise vaccination therapy, oncolytic viruses, immune checkpoint inhibition, and CAR T cell therapy. However, there are many hurdles to overcome when applying immunotherapy.

Pediatric CNS malignancies generally exhibit an immunologically “cold” phenotype, lacking infiltrating T and natural killer (NK) cells (15–17). The pathophysiology behind the absence of T cell infiltration has not been fully elucidated. However, the low tumor mutational burden, deficiencies in antigen presentation on cells, and T cell homing to the tumor bed potentially play an important role (18). Additionally, tumor-associated microglia and macrophages (TAMs), cells of the innate immune system, are the most abundant immune cell type in the tumor immune microenvironment (TIME). In healthy control brain tissue biopsies, microglia are the dominant myeloid-derived cell type in the brain. During disease, microglia are activated, and bone marrow-derived macrophages are recruited to the tumor site, where they polarize towards different activation states ranging from classically activated (M1) to alternatively activated (M2) phenotypes (19–21). They can secrete inhibitory cytokines (such as TGF-B and IL-10) promoting immunosuppression and malignant proliferation (22, 23). Macrophages can also secrete endothelial growth factors that stimulate angiogenesis, thus advancing tumor growth and metastatic invasion into surrounding tissues (24).

Cells of the TIME interact with the tumor through paracrine signaling, which can stimulate tumor progression and enable immune escape. Moreover, in other solid tumor types, different components of the TIME may function as a predictive marker of response to immunotherapy (25); therefore, characterizing the TIME, as well as other immune compartments [e.g., blood, cerebral spinal fluid (CSF), and bone marrow], is necessary for evaluating current immunotherapies and clinical implementation. Comprehensive immune monitoring programs are needed for a better understanding of the TIME, but also to find surrogate markers in blood that can be helpful in designing future immunotherapies. Currently, literature about this association is lacking.

**Figure 1:**
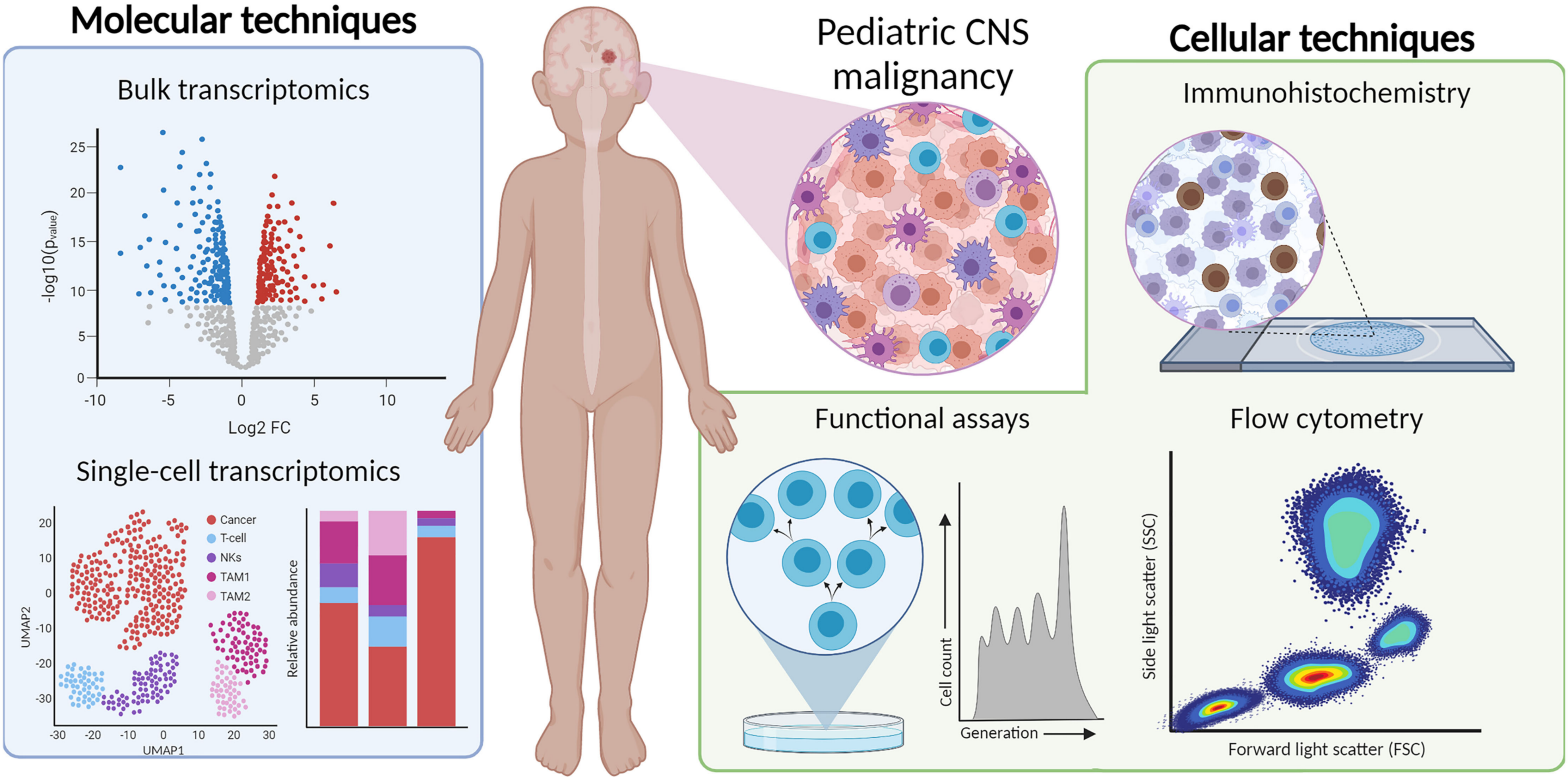
Multi-dimensional immunophenotyping of TIME from tumor tissue using IHC, flow cytometry, bulk- and scRNA-seq, and functional assays.

In this review, we provide an overview of different cellular and molecular profiling techniques for a multidimensional characterization of the immunophenotypes in pediatric CNS malignancies. We focus on immunohistochemistry (IHC), flow hen implemented simultaneously, these techniques characterize and quantify the immune cells in the microenvironment and provide detailed information into immune-status and function (**Figure 1**). We aim to discuss the advantages and pitfalls of these technologies to study the immune landscape at time of diagnosis and throughout therapy. Understanding the subtype-specific inflammatory milieu of pediatric CNS malignancies will advance our knowledge when evaluating current immunotherapies, designing new treatments, and predicting the clinical responses of patients, ultimately laying groundwork for a more personalized and safe treatment design.

## Immunohistochemistry

### Introduction to Immunohistochemistry

IHC is an essential method for detecting, quantifying, and localizing a specified protein in tissue with antibody–antigen interactions. Since its introduction in the 1970s, the clinical diagnostics field rapidly advanced wherein pathologists can better classify tumors based on their expression of lineage-specific markers (glial tumors selectively expressing GFAP), oncogenic somatic mutations (oligodendrogliomas express IDH1 R132H mutation), and epigenetic modifications (diffuse midline gliomas show H3K27me3 loss) (26, 27).

IHC is performed on formalin-fixed paraffin-embedded (FFPE) tumor slides, which allows for a retrospective analysis of preserved tissue. Antibodies are used in conjunction with a coloring dye to visualize and detect the antigen of interest on the tissue section. In the clinical diagnostics setting, automated IHC machines have standardized this process, which improved reproducibility and reduced experimental biases. Both intensity and location of the stained marker can be interpreted, which is usually done by manual scoring; however, sophisticated programs and algorithms for automatic quantification are available nowadays, which reduce interpretation biases. We refer to Kim et al. who provided a detailed overview of IHC for pathologists (28).

### Characterizing the TIME of Pediatric CNS Malignancies Using IHC

**Figure 2:**
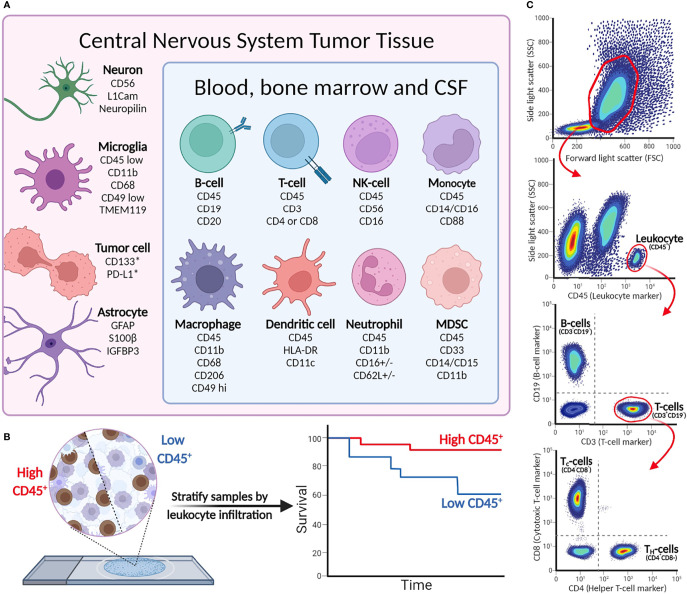
**(A)** Cell-type markers for identification of immunological, tumor, and stromal cells in CNS malignancies. **(B)** Immunohistochemistry detects leukocytes in tumor tissue, and can be used to stratify patient tumor tissue (as demonstrated in Murata et al.). *Cell surface expression may vary between tumor cells and indications. **(C)** Example of flow cytometry gating strategy. More elaborate flow panels can be used for in-depth phenotyping of immune cells, providing information on specific cell subsets and status (activation/anergic/suppressive).

In addition to the valuable applications for cancer diagnostics, IHC can provide details into the composition of the TIME. Antibodies that target antigens found on infiltrating lymphocytes, macrophages, and stromal cells are especially useful for immunophenotyping pediatric CNS malignancies (28). As IHC is an *in situ* technique, it can also describe the spatial distribution of infiltrating immune cells in the TIME. An overview of commonly used cell markers to dissect the TIME can be found in **Figure 2**.

Multiple studies used IHC to characterize the TIME of pediatric CNS malignancies, which shed unprecedented light on the composition of the TIME and revealed tumor type-specific immunophenotypes (17, 29, 30). The results demonstrated that myeloid cells are major components of the TIME of pediatric gliomas, encompassing infiltrating macrophages, myeloid-derived suppressor cells (MDSCs), and microglia, whereas there are few infiltrating lymphocytes (17, 29, 31). Analysis of the immune cell infiltration in pediatric gliomas shows an enrichment of CD8^+^ T cells and CD45^+^ leukocytes in low-grade compared to high-grade gliomas (17). In a similar way, Lieberman et al. use IHC to study immune cell infiltration in pediatric low-grade gliomas, high-grade gliomas, and diffuse midline glioma tumor samples. While the percentage of tumor-associated CD68^+^ macrophages is comparable across indications, diffuse midline gliomas have the lowest number of infiltrating CD8^+^ T cells and CD163^+^ macrophages, which contributes to its immunosuppressive phenotype (29). Moreover, multiple studies have described the subgroup-specific TIME composition of medulloblastomas. The SHH subgroup are the most enriched in CD163^+^ macrophages, suggesting different roles of tumor-associated macrophages in medulloblastoma subgroups (32). Comparing immune cell infiltration in the peritumoral area and tumor core of glioblastomas showed that CD163^+^ cells were more abundant in the tumor core. Similarly, the expression of the immunosuppressive markers PD-L1, IDO, and TIGIT was higher in the tumor core (31).

Immune profiling using IHC can be predictive of prognoses and help inform treatment strategy by detecting targets of immune checkpoint therapies, such as PD-L1. Pediatric ependymomas with higher infiltration of CD3^+^ and CD8^+^ T cells in the microenvironment at diagnosis have a longer progression-free survival (PFS), while elevated FOXP3 regulatory T cells and CD68^+^ macrophages correlate with a shorter PFS. Patients in this cohort were treated according to standard of care consisting of surgery only (59.5%), adjuvant radiotherapy (32.8%), and chemotherapy (14.6%) (33). For medulloblastoma, an increased infiltration of CD8^+^ T cells and decreased PD-L1 expression are correlated to PFS (34); however, the prognostic relevance between lymphocyte infiltration and medulloblastoma survival has been disputed (35). Other studies have quantified the presence of other checkpoint proteins, such as B7-H3 and CD155, and found that they vary largely based on cancer type (36–38). In summary, IHC is integral for characterizing the TIME in a spatial context and patient-specific manner.

### Advances in Immunohistochemistry and Spatial Proteomics

One of the major drawbacks of using IHC to characterize the TIME is the limited number of antibodies that can be used on a single tumor slide, which hampers evaluation of numerous immune cell subsets on a single slide. This is even more relevant when describing functionally active subsets of immune cells, or co-localization of antigens on tumor or immune cells. Over the last few years, more advanced techniques—such as multiplex IHC, multiplex immunofluorescence, and spatial proteomics—have been developed to study the complex spatial architecture of tissues. These methods allow for simultaneous visualization of multiple proteins, allowing to study the spatial distribution and co-localization of immune cells in the TIME in much greater detail (39, 40). Conceptually, multiplexed immunofluorescence is similar to IHC, though each antibody is labeled with a fluorophore with a unique excitation wavelength, which allows for multiple targets to be visualized on one tumor slide. Interestingly, one study designed a panel of 18 antibodies to study the effect of oncolytic virus therapy on the distribution and functional states of immune cells in a pediatric patient with glioblastoma (41). They noticed a significant increase of CD8^+^ T cells, macrophages and microglia in post-treatment tissue compared to pre-treatment tissue. Moreover, T cells expressed higher levels of CTLA-4 and PD-1 after treatment, suggesting a potential role of checkpoint inhibition. However, multiplex immunofluorescence uses frozen tissue slides that often have poorer morphology, making spatial interpretation more difficult.

Therefore, current spatial profiling approaches are based on antibodies linked to photocleavable oligonucleotide tags (such as GeoMx^®^ Protein Assays of NanoString), or use an agonistic approach by detection of isotope-labeled antibodies with mass spectrometry (42, 43). Other methods use a combination of DNA-conjugated antibodies and multicyclic addition of complementary fluorescently labeled DNA probes (44). A significant advantage of spatial proteomics over other protein-detection methods is the ability to spatially profile and quantify the high-dimensional composition of the TIME with high resolution; for example, CODEX can visualize up to 60 antigens on a single slide (44, 45). Using multiplex IHC and spatial proteomics to study the TIME of pediatric CNS malignancies could greatly enhance our understanding of the spatial composition and interactions between cells in a high-throughput manner using only a single tissue slide. To summarize, IHC is considered a highly reliable and robust method to detect protein expression and is often used to study the TIME composition.

## Flow Cytometry

### Introduction to Flow Cytometry

Since its invention in the 1960s, flow cytometry has been widely applied to characterize and quantify immune cells (46). Whereas IHC provides information on *in situ* protein expression, flow cytometry measures the expression of surface and intra-cellular proteins at the single-cell resolution. Using panels of fluorophore-conjugated antibodies directed against specific proteins, distinct (sub)populations of cells can be characterized and quantified based on their unique protein expression profiles.

Flow cytometry is used to profile various types of patient materials, such as blood, bone marrow, cerebral spinal fluid, and tissue samples once processed in a single-cell suspension. A viable single-cell suspension can be obtained from solid tissue by enzymatic digestion and/or mechanical dissociation (47–49). It is important to select a tissue-dissociation method that retains cell viability, as dead cells interfere with analysis due to their increased autofluorescence and non-specific binding of antibodies (50). Dead cells can be excluded from analysis by incorporating a specific live/dead marker in the antibody panel (50–52).

Cell handling with either freeze–thawing cycles or a Ficoll gradient to select mononuclear cells results in a loss of specific cells, and therefore, it is essential to standardize methods when initiating an immune monitoring program. Granulocytic cells are generally lost upon a freeze–thaw cycle, whereas mononuclear cells are more stable. Red blood cells are often lysed as they may interfere with the fluorescence readout (53, 54). With recent technical advances in flow cytometry, spectral flow cytometers can now analyze over 40 different extra- and intracellularly markers simultaneously.

### Characterization of Immune Cells Using Flow Cytometry

Based on the size and granularity, immune cells can be distinguished from the generally larger non-immune cells and tumor cells that are present in tumor material. Subsequently, cells can be further profiled based on the light emitted by the different cell-bound fluorophores, showing a unique phenotype for each single cell detected. An overview of the most relevant cell-specific lineage markers to discriminate subsets of leukocytes and tumor cells is given in **Figure 2**. CD45 is a general marker to identify hematopoietic cells (except erythrocytes and plasma cells). Cell-type-specific surface markers can be used for further subtyping; for example, CD3 is a lineage marker for T cells that can be further divided into CD4^+^ and CD8^+^ T cells, while CD19 is a part of the B-cell receptor complex and thus identifies B cells. The extensive multi-color options of flow cytometry allow analyses of surface-expressed markers for differentiation [naïve (effector), memory, etc.], activation [i.e., CD69 and CD137 (4-1BB)] and terminal differentiation/exhaustion (e.g., CTLA-4 and PD-1), and intracellular markers for proliferation (Ki67), production of effector molecules (cytokines/granzymes), or transcription factors (e.g., FoxP3 to define regulatory T cells). Intracellular staining requires fixation to retain cell structure and prevent intracellular proteins from diffusing out of the cells, followed by permeabilization to allow antibodies to enter the cells (55). To analyze intracellular cytokine production, cells require *ex vivo* stimulation with, for instance, phorbol myristate acetate (PMA; a NF-κB activator) and ionomycin for several hours, coinciding with Golgistop to prevent cytokine secretion, prior to performing antibody staining procedure. Detecting pro- or anti-inflammatory cytokines in the cells then provides insight into the activity of individual cells in the TIME. In conclusion, different combinations of both extra- and intracellular stains are useful for in-depth characterization of different subpopulations of cells present in TIME and periphery.

### Applications of Flow Cytometry to Pediatric CNS Malignancy Research

Though flow cytometry is not generally used for diagnosing CNS malignancies, studies have used this technique to characterize the abundance of immune cell infiltration across multiple pediatric CNS malignancies. Griesinger et al. showed differences in immune infiltration and the degree of immune suppression on biopsy material, in patients diagnosed with pilocytic astrocytoma, ependymoma, glioblastoma, and medulloblastoma (56). Compared with glioblastoma and medulloblastoma, pilocytic astrocytomas and ependymomas had significant myeloid (characterized as CD45^+^CD11b^+^) and lymphocyte infiltration. Infiltrating immune cells were shown to express low levels of PD-1, which was suggested to represent a more permissive TIME for immunotherapy. However, the authors did not investigate any additional activation markers such as CD69 or CD137. Remarkably, PD-1 expression was significantly decreased on CD4^+^ and CD8^+^ T cells in the TIME of all indications, except glioblastoma. These data were later confirmed by Plant et al. Additional flow cytometric analysis of low- and high-grade glioma, atypical teratoid rhabdoid tumor, and medulloblastoma demonstrated a trend towards increased (activated) B-cell infiltration in high- versus low-grade gliomas. Interestingly, flow analysis of peripheral blood showed low absolute lymphocyte counts in both low- and high-grade tumors, regardless of steroid treatment, which could indicate an immunosuppressive effect induced by the tumor (17).

Importantly, we are beginning to appreciate how the immunophenotype of pediatric CNS malignancies differs significantly from that of adults. Flow cytometric analysis of adult gliomas and brain metastases revealed that *IDH* mutation status and tumor origin are important in shaping the TIME. Compared to brain metastases, gliomas exhibited lower lymphocyte counts, and higher composition of microglia- and monocyte-derived infiltrating macrophages. Moreover, glioblastomas with *IDH*-wt status exhibited more lymphocyte and less macrophage infiltration compared to lower-grade *IDH*-mut gliomas (57). This correlation between tumor grading and lymphocyte infiltration has not been found in pediatric subtypes. Moreover, flow cytometric analysis demonstrated that tumor immune infiltrate in pediatric CNS malignancies does not correlate with tumor grade, as seen in adults. Low-grade gliomas have more lymphocyte and myeloid infiltration than higher-grade brain tumors (17, 56). These findings highlight that simply deducing results obtained from adult brain tumor research may not necessarily lead to the design of effective immunotherapies for children.

In addition to characterizing the TIME upon diagnosis, flow cytometry is readily used in clinical care for monitoring health and disease in various hematological indications. In hemato-oncology, immune monitoring with flow cytometry is performed on blood, CSF, or bone marrow to detect disease progression, recurrence, and to survey systemic effects of therapeutics (58, 59). However, in the context of CNS malignancies, repetitive tumor biopsies are uncommon. Studying more accessible immune compartments of solid tumors (such as blood, CSF or bone marrow) could provide valuable information on changes in the immune landscape and functional dynamics in a patient, which would otherwise be overlooked and may correlate to the TIME. To this note, a study using a glioblastoma mouse model showed evidence of glioblastoma-induced homing and accumulation of T cells (both CD4^+^ and CD8^+^) in the bone marrow. This T cell sequestration resulted in mice suffering from peripheral blood lymphopenia, a symptom that is also observed in patients diagnosed with glioblastoma (60). They also showed that T cell sequestration is a result of location rather than tumor origin, by introducing tumors of different histology in either brain or flank of the mice. Interestingly, all intracranial tumors induced T cell accumulation in the bone marrow regardless of tumor type, which was not found for subcutaneous xenografts (60). A flow cytometric and CyTOF study of adult glioblastoma patients found that MDSCs, but not regulatory T cells, are increased in the blood of glioblastoma patients compared to other CNS tumor indications. Additionally, they showed that the immune composition of blood changes during course of treatment, with reduced B cells and increased CD8^+^ T cells, dendritic cells, and MDSCs in the blood at 2 months after surgery. Finally, they showed that MDSC levels correlate with overall survival, indicating MDSCs as a possible target for immunotherapy (61). These studies provide strong evidence why investigation of other less invasive immune compartments is essential. Longitudinal flow cytometric analysis of these compartments will not only provide information on disease progression but could also be used as a tool in predicting immune treatment responsiveness.

## Bulk Transcriptomics

### Introduction to Bulk Transcriptomics

Gene expression profiling has become an indispensable tool in translational cancer research. In oncology, expression of genes provides valuable insights into the biological processes and pathways that are regulated in a tumor. For CNS malignancies, the transcriptome enables tumor subgrouping and is gaining interest as a standard tool for detection of driver mutations (62–64).

Procedures for quantifying specific RNA transcripts were first introduced with quantitative reverse transcription polymerase chain reaction (qRT-PCR), which is a robust and highly reproducible method to measure RNA expression (65). The subsequent development of microarrays allowed for the quantification of thousands of gene transcripts at the same time (66). Microarrays are based on hybridization of fluorescently labeled cDNA transcripts (obtained by reverse transcription of RNA from tissue) to complementary DNA probes (67). Nowadays, multiplex, fluorescence-based hybridization methods, such as the NanoString nCounter^®^ platform, are also frequently used to study gene expression. These techniques are based on direct detection of mRNA or miRNA from tissue and permit the use of lower-quality RNA (68, 69). A particularly interesting panel for studying the TIME is the nCounter^®^ Pancancer Immune Profiling Panel, which contains 770 genes associated with immune functions and tumor-specific antigens, spanning 24 immune cell types (70). However, these platforms only permit the quantification of a pre-defined panel of genes, and therefore the discovery of novel biomarkers is limited.

High-throughput whole transcriptome RNA-seq (from here on, referred to as “RNA-seq”) has largely replaced microarrays for gene expression profiling in onco-immunology (71). The vast amount of data that can be generated with RNA-seq from a single tumor sample has revolutionized the oncology and clinical research field (66, 72). This technique uses next-generation sequencing technologies and allows for unbiased characterization of the complete transcriptome. Briefly, RNA is isolated from fresh, frozen, or FFPE tumor tissue using preparation kits, followed by ribosomal RNA depletion. Total RNA (including mRNA, lncRNA, and miRNA) undergoes reverse transcription to convert it to cDNA libraries, which are subsequently fragmented, amplified by PCR, and sequenced (73). The sequence reads are then aligned to a reference genome and expression counts can be determined or other analyses can be performed. In contrast to hybridization-based approaches, RNA-seq can detect isoform and splice variants, and identify clinically relevant gene fusions, which are common oncogenic drivers in pediatric CNS malignancies, such as *KIAA1549::BRAF* in pilocytic astrocytomas and *C11orf95::RELA*, primarily in supratentorial ependymomas (64, 74, 75). Additionally, as prospective selection of genes is not required, the resulting expression profile is unbiased, and discovery of novel biomarkers and transcripts is possible.

### Applications of Bulk Transcriptomics to CNS Malignancy and Immune-Oncology Research

The most straightforward analysis of transcriptomic data is comparing the expression of individual genes between samples or diagnoses. For example, Lieberman et al. measured RNA expression of chemokines, pro-inflammatory cytokines, and immunosuppressive factors across pediatric gliomas and normal brain tissue. Low-grade gliomas highly express *CCL2-4*, high-grade gliomas express *CCL5*, while diffuse midline gliomas have basal expression of all chemokines (29). These results are consistent with previous studies that used IHC and flow cytometry, which described the increased lymphocyte and myeloid chemoattraction in pediatric low-grade gliomas (17, 56). Similarly, a signature, or panel of genes, can be created to study the presence and activation state of specific immune cells. These gene expression panels can incorporate functional exhaustion markers or immune checkpoints (e.g., *PD-L1*, *CTLA-4*, and *LAG-3*), to explore the possibility of targeting those with immunotherapy in pediatric brain cancers. Several tools have been developed [e.g., MCP-counter (76)] to compare the abundance of immune cell populations, based on the expression of immune-cell specific genes, between samples.

More sophisticated computational methods can be used to robustly analyze RNA-seq datasets and derive biologically meaningful results, such as targetable genes or pathways implicated in cancers. Unsupervised clustering and dimensionality reduction are useful for observing the inter-sample variability in the transcriptome. Hierarchical and k-means clustering are popular methods for identifying groups of samples with similar global gene expression patterns (77). Dimensionality reduction, such as principal component analysis or uniform manifold approximation, reduces the number of features by transforming gene expression variables into a lower-dimensional space. This retains meaningful properties of the original high-dimensional dataset and can detect heterogeneity or confounding variables among samples (78). Groups of samples identified through these approaches can be clinically relevant, as transcriptomes have been shown to correspond to anatomical location [for pilocytic astrocytomas (79, 80)], diagnosis subgroups [for medulloblastoma (81)], oncogenic drivers [H3K27 mutation status for pediatric high-grade gliomas (82)], and survival [adult and pediatric high-grade gliomas (83)].

**Figure 3:**
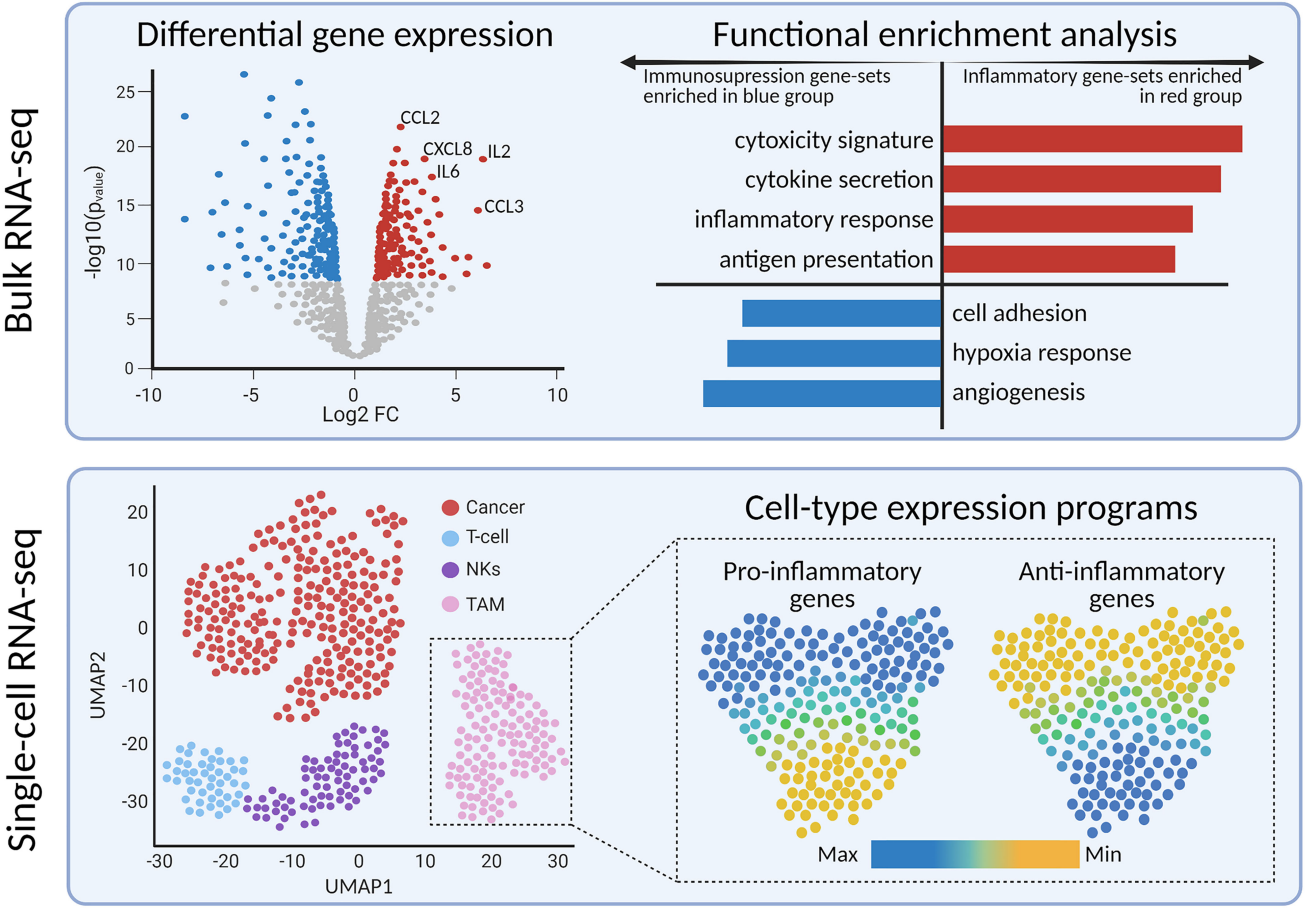
Bulk and single-cell transcriptomics reveals differentially expressed genes and pathways between tumor sub-types or cell sub-populations, respectively.

Methods such as differential gene expression and gene-set enrichment analysis provide a deeper perspective on the implicated genes and biological pathways, respectively (**Figure 3**). Differential gene expression analysis identifies quantitative differences in gene expression between two or more predefined groups; frequently used algorithms are DESeq2 (84) and edgeR (85). Differentially expressed genes can serve as diagnostic or prognostic biomarkers (83, 86). The next step is usually to translate the findings from differential gene expression analysis into pathways or gene sets to further denominate the biological processes involved. Functional enrichment analysis is useful for quantifying the expression of gene sets within groups of samples (87). There are multiple databases of gene sets, including Kyoto Encyclopedia of Genes and Genomes (88) and Molecular Signatures Database (89). In the context of immune-oncology, enrichment analysis has the potential to quantify the expression of inflammatory, cytotoxicity, or angiogenesis-related pathways. High-grade gliomas driven by alterations in MAPK pathway showed enrichment of immune-response pathways, including elevated M2-macrophage and CD8^+^ T cell signatures, compared to non-MAPK altered gliomas (90). Furthermore, gene-set analysis resolved medulloblastoma subgroup-specific TIME differences. Bockmayr et al. found that tumors of the SHH subgroup highly expressed genes related in fibroblasts, macrophages, and T cells compared with other subgroups (91).

While RNA-seq on whole tumor samples can give a broad overview of immune cell status, it is impossible to quantify changes in the individual components and cell populations of the TIME. Studies have integrated the isolation and sorting of immune cells by FACS with RNA-seq, to study transcriptional programs in cell populations. In this way, Lin et al. compared expression profiles of tumor-associated macrophages, by selecting CD11b^+^/CD45^+^ cells, of adult glioblastomas and pediatric diffuse midline gliomas. They observed a notably different gene expression profile, in which diffuse midline glioma-associated macrophages expressed lower levels of inflammatory genes (e.g., *IL6* and *CCL4*) compared to glioblastoma-associated macrophage*s* (92). In another study, macrophages and microglia from adult *IDH*-wt and *IDH*-mut glioma showed a disease-specific enrichment of inflammatory pathways: *IDH*-wt macrophages were enriched in gene sets associated with antigen presentation, and MHC I and II presentation (57). Mononuclear immune cells can also be isolated from the blood or CSF for transcriptomic profiling to define genes and gene sets implicated in various immune cells (93).

Furthermore, miRNA and lncRNA levels can also be measured with RNA-seq. The importance of non-coding RNA types in development and progression of cancer is gaining attention, both as biomarkers and as potential therapeutic targets (94). By integrating RNA-seq and miRNA expression profiling on glioblastoma tissue, Yeh et al. found that miR-138 is downregulated in glioblastomas. Subsequent *in vitro* experiments demonstrated that miR-138 downregulates CD44 expression (95). Other studies have also shown the differential expression of miRNAs or lncRNAs in brain cancers (96). While measuring miRNA and lncRNA levels with RNA-seq is still in its infancy, further studies should delineate the critical roles these RNAs play in oncogenesis and how they could be optimally targeted.

Finally, RNA-seq can further be integrated with whole-exome DNA sequencing to discover tumor-specific neoantigens. These neoantigens can be targets for tumor vaccines (97, 98). Although pediatric CNS malignancies are believed to have a low mutational burden, and therefore, a lack of targetable neoantigen expression, deep exome and transcriptome sequencing can still predict the presence of neoantigens (99, 100). For example, Rivero-Hinojosa et al. employed a multi-omics approach to detect immunogenic tumor-specific neoantigens in pediatric medulloblastomas (101). Another study detected neo-antigens in paired primary and recurrent adult glioma samples. While there was no significant difference in the amount of neoantigens between primary and recurrent samples, the expression of neoantigens was reduced at recurrence, which indicates that gliomas downregulate expression of neoantigens to evade immune recognition (102). Two phase I trials of adult glioblastoma patients have already demonstrated that personalized neo-antigen vaccines can elicit neoantigen-specific CD4^+^ and CD8^+^ T cell responses (103, 104). These findings highlight the potential of neo-antigen discovery and monitoring, which paves the way towards personalized immune cell therapy.

## Single-Cell Transcriptomics

### Introduction to Single-Cell RNA Sequencing

Whereas bulk RNA-seq averages the expression of all cells in a sample, the unique advantage of scRNA-seq is the ability to profile the transcriptome of thousands of individual cells. This technique has proven advantageous to characterize the expression programs and cell states of neoplastic and microenvironmental cells in primary and recurrent CNS malignancies (105, 106). Moreover, scRNA-seq profiling has been performed on CSF and blood to monitor the dynamic changes of leukocytes and myeloid cells during treatment (107, 108).

It is important to consider the technical and methodological challenges when designing scRNA-seq studies. First, during sample preparation, single cells must be isolated and lysed separately before sequencing, similar to flow cytometry. Tissue dissociation by enzymatic digestion can result in the loss of rare and vulnerable cell types, which would introduce experimental bias. Moreover, rare populations may not be detected as 10,000 cells are profiled in one scRNA-seq experiment. Single-nucleus RNA-seq (snRNA-seq) was developed to sequence tissues that cannot be easily dissociated into viable single-cells, including frozen tissue. snRNA-seq seems especially useful for research institutions with an extensive biobank, thus enabling a retrospective snRNA-seq on samples that were cryopreserved. Slyper et al. published a toolkit for sequencing fresh and frozen tumor samples using sc- and snRNA-seq, respectively, with recommendations for pediatric high-grade gliomas: CHAPS detergent with salts and Tris for nuclei dissociation; sequencing with the Chromium platform (109). For the purposes of simplicity in this review, we will refer to both single cell and single nucleus as “scRNA-seq”.

Additionally, researchers must decide upon sequencing technology. Full-length transcripts [produced by plate-based platforms such as Smart-Seq2 (110), RamDA-seq (111), and MATQ-seq (112)] are useful for detecting lowly expressed mRNA, splice variants, and isoforms. Droplet-based platforms [such as Chromium, DROP-seq (113), and inDrop (114)] mark the 3’ end of mRNA with a 10 base-pair unique molecular identifier (UMI); this allows for higher throughput of cells at the cost of lower resolution, which is more suitable for complex tissues with rare cell types. Batch effect from tissue type, sample preparation protocols, or research facility can be a source of confounding variation. Computational workflows may be able to correct for these uncontrolled variations (**Table 1**).

**Table 1:** Comparison of single-cell RNA-seq technologies.

*Sequencing type*	*Procedure*	*Tissue compatibility*	*Platforms*	*Advantages*
*Full-length transcripts*	Plate based: single cells are flow sorted into individual wells before library preparation	Single-cell (fresh) and single-nuclei (fresh and frozen)	Smart-Seq2 (most popular); also MATQ-seq, RamDA-seq	Captures more genes per cell; can detect splice variants and isoforms
*Unique molecular identifier (UMI)*	Droplet based: cells are encapsulated into a gel bead and 3’ or 5’ end of mRNA is marked with UMI	Single-cell (fresh) and single-nuclei (fresh and frozen)	Chromium (most popular); also DROP-seq, inDrop	Captures more cells per sample; can detect rarer cell types

### Applications of scRNA-seq to CNS Malignancy and Immuno-Oncology Research

With advances in sequencing technologies and computational biology, dimensionality reduction and clustering can characterize the diversity of cell populations (115, 116). Prior knowledge of marker genes and cell types is needed to annotate clusters within these datasets, though published packages have been developed to annotate cell types automatically. ScRNA-seq has been utilized for describing the malignant programs and developmental origins of pediatric CNS malignancies (105, 117–120).

Detailed scRNA-seq studies have also been useful for appreciating the heterogeneity and functional differences in brain residing macrophages, as some subtypes play an immunosuppressive role in high-grade gliomas (121). Compared to control brains, diseased brains with epilepsy or tumors have a greater diversity in TAM phenotypes. This suggests that TAMs functionally specialize or differentiate in brain pathologies, which was confirmed in mouse models of adult CNS malignancies (122–124). Pro-tumor and anti-inflammatory macrophages are more abundant in *IDH*-wt gliomas than *IDH*-mut, which is consistent with the poorer survival associated with *IDH*-wt glioblastoma (121). Chen et al. identified that the *MARCO* gene (macrophage receptor with collagenous structure) is selectively expressed in TAMs of *IDH*-wt glioblastomas; interestingly, *MARCO* was not expressed in TAMs of low-grade gliomas or *IDH*-mut glioma (125).

Additionally, the infiltration of TAMs and T cells has been described across various brain regions. Reitman et al. profiled the TAMs in childhood pilocytic astrocytomas using scRNA-seq (126). They found that compared to infra-tentorial pilocytic astrocytomas, supra-tentorial tumors are enriched in microglia. This provided strong evidence for differences in the glioma TIME based on anatomical location. Altogether, future studies should comprehensively characterize the contribution of macrophages and microglia to the TIME, to understand how the tumor location and genomic aberrations affect the immunophenotype.

Further characterization of the expression profiles and surface receptors of T cells have shed light into how these infiltrating lymphocytes are activated in brain cancers. As the T cell receptors (TCRs) mediate T cells’ response to cancer, computational methods have been developed to sequence the TCRs to understand how these cells proliferate and specialize (127). ScRNA-seq reads can be used to computationally reconstruct the TCR chains. These powerful methods allow for simultaneous measurements of the transcriptional profile and TCR of T cells from scRNA-seq data (128–130). A recent study by Mathewson et al. extensively profiled the transcriptomes and TCRs of infiltrating T cells in *IDH*-wt glioblastomas and *IDH*-mut high-grade gliomas (131). CD8+ and CD4+ T cells in IDH-wt glioblastoma had higher expression of cytotoxicity, interferon, and cellular stress pathways than *IDH*-mut gliomas. Additionally, they used TraCeR, a computational method to reconstruct the TCRs, to describe the clonal expansion of T cells upon infiltration. On average, each tumor contains 13 distinct T cell clonotypes, which provides evidence for T cell activation and expansion in high-grade gliomas. Of note, more clonal CD8^+^ T cells have higher expression of reactivity and cytotoxicity signatures, which was confirmed by Rubio-Perez et al. (108). Overall, these analyses highlight the utility of scRNA-seq to investigate transcriptional programs and expansion of T cells in adult brain cancers. However, it may be challenging to profile TCRs in pediatric CNS malignancies as they typically exhibit low levels of T cell infiltration. Therefore, it would be necessary to first sort for CD3^+^ T cells, thereby excluding the neoplastic, TAMs, and other infiltrating immune cells present in tumor tissue (131).

Overall, scRNA-seq has the potential to identify transcriptional changes in the neoplastic and microenvironmental cells throughout treatment, which is reliant on longitudinal sampling. Ruiz-Moreno et al. monitored the expression profiles of microglia, macrophages, and T cells in a patient with a diffuse midline glioma at diagnosis and metastasis (132). Upon biopsy, the immune component made up half of the primary tumor, mostly consisting of microglia and a few macrophages. These tumor-associated macrophages expressed an anti-inflammatory/pro-tumor phenotype, which supported their finding of few infiltrative T cells. Three serial samples of the abdominal tumor metastasis were obtained; interestingly, they found that, over time, macrophages obtained a more pro-tumor phenotype with few infiltrating pro-inflammatory macrophages or T cells. Finally, the researchers also used scRNA-seq to describe changes in the TIME after the patient started treatment with hydroxychloroquine, an immunomodulator that increases the anti-tumor activity of macrophages (132). Their analysis revealed a dramatic increase in naïve and pro-inflammatory macrophages, and antigen-presenting dendritic cells. Their results highlight the utility of longitudinal sampling and scRNA-seq to understand the evolution of the TIME throughout disease course and immunotherapies.

Due to difficulties with re-sampling primary tumor tissues, researchers have used scRNA-seq to characterize other compartments of the immune system, including CSF and peripheral blood (108, 133). However, the question remains if the immune cells in the CSF or blood are informative for the brain TIME and should be further elucidated in future studies. In a study of adults with brain metastasis, Rubio-Perez et al. obtained simultaneous scRNA-seq datasets of brain metastasized tumor and CSF (108). The TIME and CSF had consistent proportions of CD8^+^ T, CD4^+^ T, and NK cells, highlighting how scRNA-seq data of lymphocytes in liquid biopsies recapitulate the TIME of primary tumors (108). As the TIME is correlated to patient’s response to immunotherapies, future studies need to explore if liquid biopsies do reflect the TIME in pediatric brain malignancies, with the ultimate goal to have a surrogate for response to (immune) therapy (25). However, not all compartments of the immune system behave similarly. Prakadan et al. also obtained scRNA-seq datasets of peripheral blood and CSF of patients enrolled in clinical trials for immune checkpoint inhibitors for leptomeningeal disease (133). Lymphocytes and myeloid cells expressed gene signatures for antigen presentation and IFN-gamma response higher in the CSF compared to the blood. They also found that myeloid cells in the CSF showed an increase in the pro-inflammatory phenotype, while the opposite was seen in myeloid cells in the blood.

**Figure 4:**
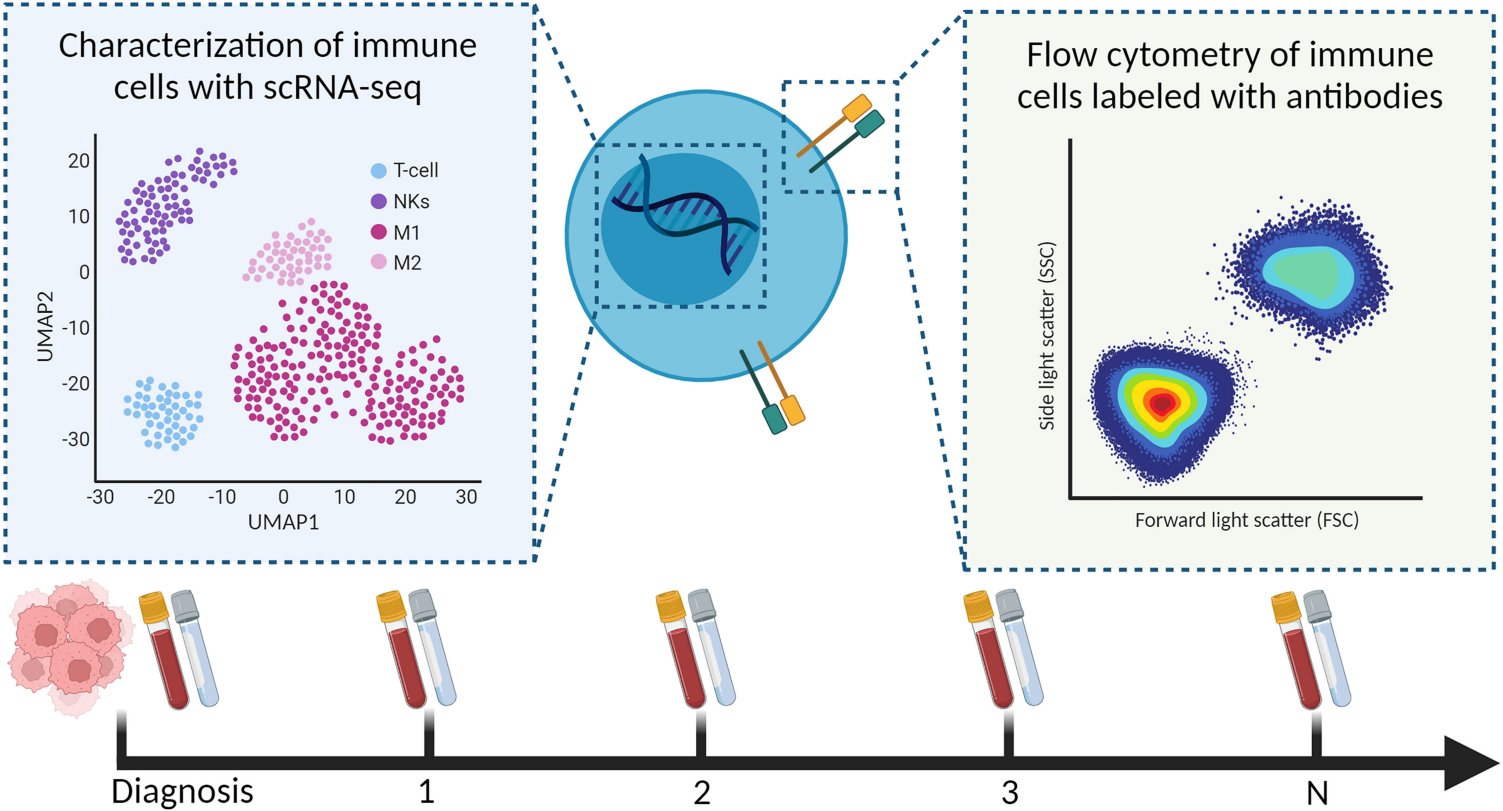
Characterization of immune cells from longitudinal liquid biopsies (as shown, blood and CSF) using scRNA-seq and flow cytometry.

Longitudinal samples of liquid biopsies are useful when relating immune system dynamics with clinical outcomes. Soon after treatment with checkpoint inhibitors, Prakadan et al. identified an increased inflammatory gene signature in the CSF, which was not identified at later time points (133). Additionally, compared with pre-treatment samples, CD8^+^ T cells significantly expressed genes associated with antigen presentation and IFN-gamma signaling. These results could explain the clinical benefits of the immune checkpoint inhibitors in leptomeningeal disease. Furthermore, Rubio-Perez et al. mapped the abundance of immune cells in the CSF for two patients that underwent re-surgery; their serial samples of CSF showed an increase in naïve T cells and decrease in macrophages (108). Together, these papers provide evidence that scRNA-seq should be further investigated for children with a CNS malignancy; preferably including longitudinal sampling to understand, and hopefully improve, (immuno)therapies in pediatric high-grade brain tumors (**Figure 4**).

### Advances in Transcriptomic Profiling

With advancements in transcriptomics technologies and computational methods, we have seen a surge in creative applications using scRNA-seq to investigate the tumor ecosystem. Computational methods referred to as “deconvolution” integrate bulk and scRNA-seq, and have been developed to predict the cell-type composition from homogenized bulk RNA-seq data. Algorithms for cell-type deconvolution include CIBERSORTx, MuSiC, and DWLS (134–136). Though they differ in their mathematical methods, the results are highly robust and correlated (137). Results from deconvolution should be benchmarked with representative IHC staining or flow cytometry, to ensure that differences in RNA expression correspond to cell-type quantification from the protein and microscopy level. Additionally, cell–cell interactions have been predicted from scRNA-seq datasets of heterogeneous tissues, which is especially useful for characterizing the TIME. Simply, these algorithms utilize literature-derived datasets of ligand–receptor interactions, and predict which cells communicate based on the cell-type expression of these proteins (138). Physical location information from spatial transcriptomics or proteomics would increase the power of these data-driven predictions, as interacting cells are located near one another. Both deconvolution and cell–cell interaction algorithms will advance research studies aiming to quantify the immune cell composition and interactions within the TIME.

scRNA-seq has been adapted to profile transcriptomics in parallel with additional biomolecules. CITE-seq was developed to allow for simultaneous profiling of multiplexed protein markers and transcriptomes of single cells from fresh tissue (139). In this procedure, single cells are incubated with DNA-barcoded antibodies. Upon sequencing, a quantitative read out is obtained for the expression and immunophenotyping of single cells. CITE-seq has been used to profile the immune cells in the glioblastoma microenvironment (123). TAMs and dendritic cell clusters were identified in CITE-seq and scRNA-seq analyses, proving the robustness of the protocols. One unique benefit of CITE-seq is identifying novel cell surface protein markers; for example, markers were identified that captured monocyte-to-macrophage differentiation and characterized blood-derived macrophages from microglia (139).

Finally, as bulk- and scRNA-seq sample preparation requires tissue dissociation, spatial transcriptomics methods have been developed to retain spatial information of mRNA transcripts that is lost with other sequencing platforms (140–142). Fresh-frozen or FFPE tissues are transferred onto a slide with millions of oligonucleotide probes containing a “spatial barcode”. The RNA binds with the oligonucleotides on the slide; upon sequencing, the tissue’s RNA can be spatially resolved and mapped to histology images of the tissue. Commonly used scRNA-seq analysis tools [such as Seurat (115)] are compatible with spatial transcriptomics and allow for similar clustering analysis of distinct tumor regions. It is important to note that the resolution of spatial transcriptomics is less than that of scRNA-seq; each spatial barcode can contain between 3 and 30 cells on the Visium platform, and profiles the expression of far fewer genes. It may be more challenging to detect intra-patient heterogeneity for embryonal tumors that have malignant cells that are densely packed together.

Spatial transcriptomics seems especially useful for resolving the biology at the tumor–microenvironment interface in solid tumors. A recent study from Ravi et al. identified a subset of myeloid cells that are co-localized with mesenchymal-like glioblastoma cells that drive T cell exhaustion, thus contributing to the anti-inflammatory TIME (143). Another study characterized a unique cell state localized at the tumor–microenvironment boundary, which had upregulation of cilia gene sets (144). For heterogeneous and infiltrative pediatric CNS malignancies such as high-grade gliomas, spatial transcriptomics can radically advance our understanding of how cancer cells interact with neurons, astrocytes, and immune cells, and eventually, how the microenvironment architecture changes due to therapeutic interventions.

## Functional Assays

IHC, flow cytometry, and transcriptomics are important for describing the composition of immune cells in the TIME. The presence of proliferation markers or enrichment of inflammatory gene sets is suggestive of a cell’s potential to mediate humoral or cellular immunity. However, the true capacity of these immune cells to proliferate or kill target cells (referred to as “cytotoxicity”) can only be confirmed using functional cellular assays. To this end, tumor-infiltrating lymphocytes (TILs) can be isolated from tumor material or liquid biopsies using cell sorting selective for CD45 markers and evaluated *ex vivo*. As an alternative to single-cell preparation and sorting, TILs can also be expanded directly from tumor materials by culturing the material for several weeks in the presence of stimulatory agents (IL-2, with or without anti-CD3 stimulus) (104, 145). This method for TIL expansion has been used successfully in adoptive cell therapy in multiple cancer indications (146–148); however, it has the disadvantage that cellular diversity is lost, as these culture conditions only promote clonal expansion of a select subset of TILs (mainly T cells).

The proliferative capacity of lymphocytes, indicative for an active immune response, can be investigated by fluorescently labeling the cells with a cell tracer dye [such as cell tracer violet or carboxyfluorescein diacetate succinimidyl ester (CFSE)] and culturing in the presence of an activating agent (149). Polyclonal activation of TILs can be induced with either a lectin mitogen (i.e., phytohemagglutinin) or with anti-CD3 or CD3/CD28 beads in the presence or absence of stimulatory cytokines [i.e., IL-2 (149)]. Upon proliferation, the cell tracer dye will get divided among daughter cells and, therefore, multiple peaks will be visible with a decreased fluorescence.

Clonal activation and expansion of TILs is performed by coculturing the cells for several weeks with irradiated and peptide loaded feeder cells, in the presence of IL-2 (150). Feeder cells can be either autologous or allogeneic antigen-presenting cells (APCs) or tumor cells expressing the peptide(s) of interest (e.g., pp65 or H3K27M) on their MHC (151). The use of these feeder cells dates back to the 1980s and has shown to promote T cell culture (152). A clinical study using a similar procedure to clonally expand TILs from melanoma patients showed the presence of neo-antigen-specific T cells. Adoptive cell transfer of these TILs, supplemented with IL-2 dosing, resulted in a clinical response in 5 out of 10 patients of which 2 were complete responders (153). Additionally, brain metastases from melanoma have been shown to be susceptible to TIL therapy. In a retrospective study, 7 of 17 (41%) melanoma patients with brain metastases receiving adoptive TIL therapy achieved complete response (154). These results are promising and provide hope that TIL therapies could also be of benefit for the treatment of primary CNS malignancies, although more hurdles will need to be overcome, such as an immune-suppressive environment and the blood–brain barrier. Liu et al. demonstrated that TILs from glioma can be efficiently expanded using a combination of IL-2, IL-15, and IL-21, enhancing CD8 T cell reactivity to autologous tumor cells (155). More recently, a clinical phase I trial has started, investigating the safety and efficacy of TIL therapy in malignant glioma patients (NCT04943913).

Finally, cytokine secretion and cytotoxicity assays are essential for determining the capability of immune cells to induce direct cytotoxicity. The cytokine secretome can be measured directly from patient samples (including tumor tissue, CSF, or plasma), or from conditioned mediums of tumor cell cultures. Measuring both pro-inflammatory (e.g., IFNγ, TNFα, and IL-2) and anti-inflammatory cytokines (e.g., IL-4, IL-6, and IL-10) provides an indication of immune activity in the tumor. Several multiplex techniques such as Olink and Luminex were developed to measure over 25 different cytokines in a limited amount of material. Findings from these assays highlight the anti-inflammatory nature of pediatric CNS malignancies. The secretome of diffuse midline gliomas and pediatric glioblastomas are remarkably different to that of adult glioblastomas. Pediatric high-grade gliomas do not secrete substantial levels of inflammatory cytokines that recruit lymphocytes to the TIME, which contributes to their immunosuppressive phenotype (92). Moreover, assays wherein pediatric glioma cells were co-cultured with T and NK cells suggest therapeutic strategies for immunotherapies. T cells could not effectively lyse diffuse midline glioma cell lines, while NK cells exhibited cytotoxic effects (29). Altogether, cytotoxicity assays are essential in immune monitoring programs to validate hypotheses regarding the anti-inflammatory TIME of pediatric CNS malignancies.

## Discussion

### Towards an Immunological Atlas of Pediatric CNS Malignancies

Current neuro-oncological practice is increasingly dependent on molecular and cellular profiling of CNS tumor tissue. These histo-molecular findings contribute to a more accurate diagnosis and have the potential to direct personalized and targeted treatment, especially in the context of immunotherapies. A comprehensive evaluation of the pediatric CNS tumor microenvironment and immune system at diagnosis, across treatment and at relapse will be necessary to not only understand how the immune system responds to radio- and systemic therapies, but also to evaluate how novel immunotherapies modulate the immune system. Without aiming to discuss all available methods, we selected various techniques that provide a comprehensive atlas of multiple compartments of the immune system at diagnosis and throughout treatment. We analyzed the clinical utility and feasibility of IHC, flow cytometry, bulk and single-cell transcriptomics, and functional assays for monitoring the immune system and TIME of pediatric CNS malignancies (**Table 2**).

**Table 2:** Utility and feasibility of selected techniques for immune profiling of tumor tissue.

Technique	Tissue type	Utility	Advantages	Disadvantages
***Immuno-histochemistry***	FFPE	Quantification and phenotyping	Routine use in diagnostics Validation for other techniques Retains spatial information	Low throughput
***Flow Cytometry***	Fresh	Quantification and phenotyping	Millions of cells profiled Fast data acquisition and analysis	Panel of antigens or cell types (biased) Loss of spatial information
***Bulk Transcriptomics***	Fresh, frozen, FFPE	Biomarkers, functional pathways	Routine use in diagnostics Identify pathways for targeted treatment	Loss of spatial and cell-type information Computationally intensive
***Single-cell Transcriptomics***	Fresh, frozen	Quantification, functional pathways	Thousands of cells profiled Retains cell-type information Identify rare or novel cell types	Expensive ($2,000/sample) Time-consuming analysis Computationally intensive Loss of spatial information
***Functional assays***	Fresh	Immune-cell function	Cytotoxicity or proliferation potential of TILs *Ex vivo* and *in vitro* activity	Low throughput Reproducibility of assays

Studies of IHC are useful to determine the quantity and location of a specific cell type in the TIME. In summary, IHC is performed on formalin-fixed paraffin embedded tissue, thus retains spatial information, and enables retrospective analyses. With developments in multiplex IHC and flow cytometry, multiple cell types can be quantified to provide a more detailed atlas. Flow cytometry is beneficial as it profiles up to millions of cells in a short period of time (approximately 3 h). However, flow cytometry requires a viable suspension of millions of live single cells; therefore, the sample must be substantially larger than required for IHC or sequencing (where scRNA-seq requires a few thousand cells) and should be either viably cryopreserved or processed directly after surgery (where the latter is preferred to better characterize the myeloid components of the TIME). Additionally, antibody-based technologies require the development and assessment of a panel, which pre-selects the proteins (and therefore, cells) that can be interrogated. Another limitation is that certain cell types may have similar surface proteins compared to other cells, complicating the identification of the cells of interest (i.e., distinguishing MDSCs from myeloid cells). However, as both IHC and flow cytometry are cost-effective and readily available at almost all academic research hospitals, their utility should not be overlooked. Overall, they are useful for quantifying and phenotyping the immune cells present in the TIME; however, the analysis is hypothesis driven, and this introduces a selection bias when using a panel of antibodies. As we described, there is already much prognostic value in quantification metrics alone: for example, T cell infiltration is related to tumor grade in pediatric gliomas and progression-free survival in ependymomas (29, 33).

High-throughput genomic techniques, such as bulk and single-cell transcriptomics, quantify thousands of genes by sequencing. As these methods are unbiased (i.e., not relying on a panel of pre-selected RNA transcripts), they can be useful for identifying gene expression patterns with prognostic value. With bulk RNA-seq, evaluating the expression of immune-related genes and enrichment of immune-related pathways can reveal the inflammatory state of the TIME (29, 90). A possible, and promising, solution is to perform RNA-seq on immune cells found in blood or CSF, which could be correlated to the TIME (93, 156). Although, to our knowledge, no such experiments have been performed to monitor pediatric CNS malignancies yet, studies on other cancers have shown the potential of using these more readily accessible biomaterials to monitor the anti-tumor immune response during therapy. For example, circulating miRNA levels were found to be correlated to the absolute neutrophil count in pancreatic cancer (93). Moreover, comparing immune signatures of patients who respond to those who do not respond to a particular type of therapy can eventually guide therapy decision (157). However, the low number of tumor-infiltrating immune cells compared to the high density of tumor cells can limit the sensitivity of this technique. Furthermore, scRNA-seq can uniquely distinguish rare cell types and cell states; for example, scRNA-seq datasets of T cells in adult gliomas revealed differences in the expression of cytotoxicity and stress pathways between *IDH*-mut and *IDH*-wt tumors that could not be identified *via* IHC or flow cytometry alone (131). Though they are incredibly useful for investigating biological pathways implicated in diseases, transcriptomic methods are costly and computationally intensive. Spatial transcriptomics has been developed to retain the spatial information lost due to tissue dissociation protocols.

These cellular and molecular techniques are essential for immunophenotyping CNS malignancies. Additional functional experiments that assess the TIME cells’ cytokine secretion profile or cytotoxicity capacity of immune cells are necessary to evaluate hypotheses generated from the prior methods. Such assays helped us realize that diffuse midline gliomas have a non-inflammatory TIME by secreting much fewer cytokines than adult high-grade gliomas (92). Additionally, they revealed that NK cells can lyse diffuse midline glioma cells, while T cells and macrophages cannot (29). Results from functional assays suggest that immunotherapies that recruit and activate NK cells could be plausible strategies for new treatment designs. In summary, integrating IHC, flow cytometry, transcriptomic methods, and functional assays allow for a detailed atlas of the quantity, location, and functionality of immune cells in both TIME and periphery, which cannot be achieved by one methodology alone.

### Clinical Perspective

We recognize that there are significant challenges with designing studies that monitor the immune system during cancer treatment. Pediatric normal brain tissue is a necessary control for characterizing the infiltration and activation of immune cells in CNS malignancies but is scarce and difficult to obtain. Fresh CNS material can be obtained from epilepsy surgeries and tumor-free autopsy material (56); however, there are still confounding factors to consider, such as cause of mortality and time since death. Additionally, “brain banks” dedicated to storing and distributing postmortem brain tissues for the research community have been established worldwide, though these typically consist of adult specimens (158). The National Institute of Child Health and Human Development and the Harvard Brain Tissue Resource Center are two brain banks specific for collecting and distributing brain tissue from children and fetuses (159).

Furthermore, obtaining high-quality CNS tumor samples remains the biggest hurdle, especially for cases when the tumor is in a vulnerable location (i.e., the brain stem for diffuse midline gliomas). CUSA material (tissue obtained using a Cavitron Ultrasonic Surgical aspirator during brain surgery), which contains buffered solution, brain tumor and non-tumor fragments, and blood, can also be used for cellular and molecular analyses. CUSA material has been used for diagnostic purposes, and in studies of cellular heterogeneity of malignant cells and immune microenvironment (160–163). To what extent the CUSA material reflects the microenvironment of the tumor in children is subject to further research.

Longitudinal sampling to evaluate treatment effects is also particularly challenging. In a recent pre-print, Spitzer et al. obtained scRNA-seq datasets of two matched pre- and post-treatment glioma samples that responded well to a targeted treatment and identified changes in the malignant cell states that explained the observed clinical response. To increase the power and confidence of their analysis, they included pre-treatment scRNA-seq data of non-responders, and noticed a distinct differentiation pattern that could explain their lack of clinical response (164). Additionally, we suggest utilizing liquid biopsies to characterize the immunological system response to treatment. Liquid biopsies are currently a hot topic for assessing progression of pediatric CNS malignancies, with a recent paper demonstrating that serial analysis of circulating DNA in the CSF can predict tumor burden and disease progression in medulloblastomas (165). As we described, flow cytometry and scRNA-seq datasets of immune cells in blood, bone marrow, and CSF can also be informative for advancing our understanding of the immune system’s response to current treatment regimens and designing future immunotherapies (108, 133). Ideally, we can use liquid biopsies as surrogate markers for the TIME, avoiding invasive surgical procedures.

**Figure 5:**
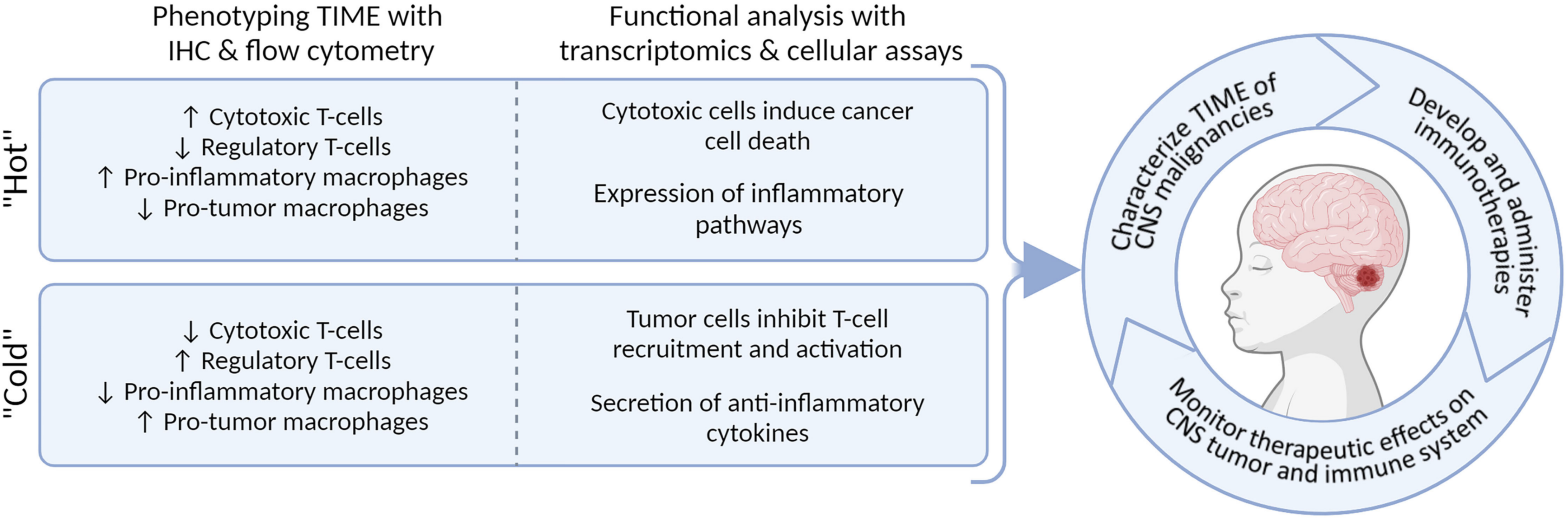
Integrating methodologies allows for multi-dimensional characterization of TIME and will enable personalized immunotherapeutic strategies.

Looking forward, we predict that a detailed description of the microenvironment and immune system of pediatric CNS malignancies throughout the disease course will be paramount to implementing and evaluating the efficacy of immunotherapies (**Figure 5**). In our field, we are beginning to recognize substantial biological differences between pediatric and adult CNS malignancies, which could also be attributed to the role of the immune system in childhood neurodevelopment. Studies of pre- and post-natal mice have been essential in describing the unique role that microglia take as modulators in the formation of neural circuits: they clear excess neurons, assist with vascularization, and regulate neural-stem cell commitment to astrocyte and neuron lineages (166). During adulthood, microglia become ramified and survey the brain parenchyma for tissue injury or disease (166). Interestingly, in the setting of brain cancers, microglia do not phagocytize the glioma cells, and can be reprogrammed to promote tumor growth (167). Detailed scRNA-seq studies of adult and mouse gliomas could resolve the heterogeneity of microglia states (121–125); however, similar studies in children are lacking but will be necessary to unravel the complex activity of microglia in malignancy and development.

In the field of pediatric neuro-oncology, trials of vaccine approaches, oncolytic viruses, checkpoint blockade, and adoptive cellular therapy are currently under investigation in preclinical and clinical settings. Small trials thus far show noteworthy clinical outcomes but raise a broad spectrum of unique challenges that need to be evaluated (168). Specific challenges while developing immunotherapies for children with CNS malignancies include a cold immunophenotype with minimal T cell infiltration, a relatively low mutational burden, intra-tumoral heterogeneity, and blood–brain barrier penetration. The potency of immunotherapy needs to be evaluated for optimal application of immunotherapy targeting this highly specific neoplasm and challenging environment. Clinical outcomes targeting a single tumor-associated antigen failed to show adequate, durable anti-tumor responses, suggesting that selective targeting of one antigen may be insufficient (168, 169). Combination therapy might help overcome tumor heterogeneity and penetrate the highly immunosuppressive TIME, and clinical trials are underway (i.e., NCT03130959, NCT00634231, and NCT03396575). Furthermore, clinical research studies should focus on elucidating the long-term side effects of immune modulators in children, especially when addressing effects to their developing immune system, and neurodevelopment and cognition.

To move forward, we need a multifaceted and comprehensive description of the microenvironment of pediatric CNS malignancies. Understanding the subtype-specific immunological phenotype and how the treatment protocol, anatomical location, and grade of each tumor influences the TIME is essential to efficiently redirect the immune system towards cancer eradication. Within the Princess Maxima Center, Utrecht, Netherlands, a prospective observational longitudinal study is initiated including 60 newly diagnosed and relapsed children with various CNS malignancies (Trial NL8967). At diagnosis, biopsy tumor tissue will be profiled by IHC, bulk, and single-cell transcriptomics. CSF, bone marrow, and peripheral blood will be collected during surgery and subsequent follow-up visits; these liquid biopsies are profiled by flow cytometry and scRNA-seq (depending on sample size and tissue availability). Accurate immune profiling could help pave the way for future immunotherapeutic interventions in pediatric neuro-oncology.

## Author Contributions

JSR, JIM-E, JASL, MLB, FGC, and JvdL wrote the review. JSR coordinated the writing and review process. SN, MEGK, and LAK critically revised the drafts. JSR, JIM-E, JASL, and MLB made the figures and tables. All authors contributed to the conception of this review and have made a substantial, direct, and intellectual contribution to the work and approved it for publication.

## Funding

This study received funding from the Team Westland Foundation. JSR was supported by the Fulbright U.S. Student Grant and Netherland-American Foundation Pediatric Cancer Award. The funders were not involved in the study design, collection, analysis, interpretation of data, the writing of this article, or the decision to submit it for publication.

## Conflict of Interest

The authors declare that the research was conducted in the absence of any commercial or financial relationships that could be construed as a potential conflict of interest.

## Publisher’s Note

All claims expressed in this article are solely those of the authors and do not necessarily represent those of their affiliated organizations, or those of the publisher, the editors and the reviewers. Any product that may be evaluated in this article, or claim that may be made by its manufacturer, is not guaranteed or endorsed by the publisher.

## References

[1] Louis DN Perry A Wesseling P Brat DJ Cree IA Figarella-Branger D. The 2021 WHO Classification of Tumors of the Central Nervous System: A Summary. Neuro Oncol (2021) 23(8):1231–51. doi: 10.1093/neuonc/noab106 PMC8328013 34185076

[2] Miller KD Ostrom QT Kruchko C Patil N Tihan T Cioffi G. Brain and Other Central Nervous System Tumor Statistics, 2021. CA Cancer J Clin (2021) 71(5):381–406. doi: 10.3322/caac.21693 34427324

[3] Packer RJ Zhou T Holmes E Vezina G Gajjar A. Survival and Secondary Tumors in Children With Medulloblastoma Receiving Radiotherapy and Adjuvant Chemotherapy: Results of Children’s Oncology Group Trial A9961. Neuro Oncol (2013) 15(1):97–103. doi: 10.1093/neuonc/nos267 23099653 PMC3534419

[4] Fangusaro J. Pediatric High Grade Glioma: A Review and Update on Tumor Clinical Characteristics and Biology. Front Oncol (2012) 2:105. doi: 10.3389/fonc.2012.00105 22937526 PMC3426754

[5] El-Ayadi M Ansari M Sturm D Gielen GH Warmuth-Metz M Kramm CM. High-Grade Glioma in Very Young Children: A Rare and Particular Patient Population. Oncotarget (2017) 8(38):64564–78. doi: 10.18632/oncotarget.18478 PMC5610026 28969094

[6] Grundy R. The Development of Cell Line Models of Childhood Brain Tumours. ATLA (2010) 38(Supplement 1):11–3. doi: 10.1177/026119291003801S09 21275477

[7] Denunzio NJ Yock TI. Modern Radiotherapy for Pediatric Brain Tumors. Cancers (Basel) (2020) 12(1533):1–16. doi: 10.3390/cancers12061533 PMC7352417 32545204

[8] Pollack IF Agnihotri S Broniscer A. Childhood Brain Tumors: Current Management, Biological Insights, and Future Directions. J Neurosurg Pediatr (2019) 23(3):261–73. doi: 10.3171/2018.10.PEDS18377 PMC6823600 30835699

[9] Miklja Z Pasternak A Stallard S Nicolaides T Kline-nunnally C Cole B. Neuro-Oncology Gliomas: Review and Consensus Recommendations. Neuro Oncol (2019) 21(February):968–80. doi: 10.1093/neuonc/noz022 PMC6682212 30805642

[10] Wolchok JD Chiarion-Sileni V Gonzalez R Rutkowski P Grob JJ Cowey CL. Overall Survival With Combined Nivolumab and Ipilimumab in Advanced Melanoma. N Engl J Med (2017) 377(14):1345–56. doi: 10.1056/NEJMoa1709684 PMC5706778 28889792

[11] Larkin J Chiarion-Sileni V Gonzalez R Grob JJ Cowey CL Lao CD. Combined Nivolumab and Ipilimumab or Monotherapy in Previously Untreated Melanoma. N Engl J Med (2015) 373(1):23–34. doi: 10.1056/NEJMoa1504030 26027431 PMC5698905

[12] Motzer RJ Escudier B McDermott DF George S Hammers HJ Srinivas S. Nivolumab Versus Everolimus in Advanced Renal Cell Carcinoma. N Engl J Med (2015) 373(19):1803–13. doi: 10.1056/NEJMoa1510665 PMC5719487 26406148

[13] Borghaei H Paz-Ares L Horn L Spigel DR Steins M Ready NE. Nivolumab Versus Docetaxel in Advanced Non-Squamous Non- Small Cell Lung Cancer. N Engl J Med (2017) 373(17):1627–39. doi: 10.1056/NEJMoa1507643 PMC5705936 26412456

[14] Vitanza NA Johnson AJ Wilson AL Brown C Yokoyama JK Künkele A. Locoregional Infusion of HER2-Specific CAR T Cells in Children and Young Adults With Recurrent or Refractory CNS Tumors: An Interim Analysis. Nat Med (2021) 27(9):1544–52. doi: 10.1038/s41591-021-01404-8 34253928

[15] Jones C Karajannis MA Jones DTW Kieran MW Monje M Baker SJ. Neuro-Oncology in Need of New Thinking. Neuro Oncol (2017) 19(June 2016):153–61. doi: 10.1093/neuonc/now101 PMC5464243 27282398

[16] Yalon M Toren A Jabarin D Fadida E Constantini S. Elevated NLR May Be a Feature of Pediatric Brain Cancer Patients. Front Oncol (2019) 9(April):1–5. doi: 10.3389/fonc.2019.00327 31114757 PMC6502986

[17] Plant AS Koyama S Sinai C Solomon IH Griffin GK Ligon KL. Immunophenotyping of Pediatric Brain Tumors: Correlating Immune Infiltrate With Histology, Mutational Load, and Survival and Assessing Clonal T Cell Response. J Neurooncol (2018) 137(2):269–78. doi: 10.1007/s11060-017-2737-9 29322427

[18] Bonaventura P Shekarian T Alcazer V Valladeau-Guilemond J Valsesia-Wittmann S Amigorena S. Cold Tumors: A Therapeutic Challenge for Immunotherapy. Front Immunol (2019) 10:168. doi: 10.3389/fimmu.2019.00168 30800125 PMC6376112

[19] Ajami B Bennett JL Krieger C McNagny KM Rossi FMV. Infiltrating Monocytes Trigger EAE Progression, But do Not Contribute to the Resident Microglia Pool. Nat Neurosci (2011) 14(9):1142–9. doi: 10.1038/nn.2887 21804537

[20] Sampson JH Gunn MD Fecci PE Ashley DM. Brain Immunology and Immunotherapy in Brain Tumours. Nat Rev Cancer (2020) 20(1):12–25. doi: 10.1038/s41568-019-0224-7 31806885 PMC7327710

[21] Murray PJ Allen JE Biswas SK Fisher EA Gilroy DW Goerdt S. Macrophage Activation and Polarization: Nomenclature and Experimental Guidelines. Immunity (2014) 41(1):14–20. doi: 10.1016/j.immuni.2014.06.008 25035950 PMC4123412

[22] Gate D Danielpour M Rodriguez J Kim G Levy R Bannykh S. T-Cell TGF- β Signaling Abrogation Restricts Medulloblastoma Progression. PNAS (2014) 111(33):E3458–E3466. doi: 10.1073/pnas.1412489111 25082897 PMC4143044

[23] Huettner C Czub S Kerkau S Roggendorf W Tonn JC. Interleukin 10 is Expressed in Human Gliomas In Vivo and Increases Glioma Cell Proliferation and Motility *In Vitro* . Anticancer Res (1997) 17(5A):3217–24. 9413151

[24] Ostuni R Kratochvill F Murray PJ Natoli G. Macrophages and Cancer: From Mechanisms to Therapeutic Implications. Trends Immunol (2015) 36(4):229–39. doi: 10.1016/j.it.2015.02.004 25770924

[25] Taube JM Klein A Brahmer JR Xu H Pan X Kim JH. Association of PD-1, PD-1 Ligands, and Other Features of the Tumor Immune Microenvironment With Response to Anti–PD-1 Therapy. Clin Cancer Res (2014) 20(19):5064–74. doi: 10.1158/1078-0432.CCR-13-3271 PMC4185001 24714771

[26] Gajjar A Pfister SM Taylor MD Gilbertson RJ. Molecular Insights Into Pediatric Brain Tumors Have the Potential to Transform Therapy. Clin Cancer Res (2014) 20(22):5630–40. doi: 10.1158/1078-0432.CCR-14-0833 PMC4234174 25398846

[27] Huang T Garcia R Qi J Lulla R Horbinski C. Detection of Histone H3 K27M Mutation and Post-Translational Modifications in Pediatric Diffuse Midline Glioma *via* Tissue Immunohistochemistry Informs Diagnosis and Clinical Outcomes. Oncotarget (2018) 9(98):37112–24. doi: 10.18632/oncotarget.26430 PMC6324678 30647848

[28] Kim SW Roh J Park CS. Immunohistochemistry for Pathologists: Protocols , Pitfalls , and Tips. J Pathol Transl Med (2016) 50:411–8. doi: 10.4132/jptm.2016.08.08 PMC5122731 27809448

[29] Lieberman NAP Degolier K Kovar HM Davis A Hoglund V Stevens J. Characterization of the Immune Microenvironment of Diffuse Intrinsic Pontine Glioma: Implications for Development of Immunotherapy. Neuro Oncol (2019) 21(1):83–94. doi: 10.1093/neuonc/noy145 30169876 PMC6303470

[30] Ott M Prins RM Heimberger AB. The Immune Landscape of Common CNS Malignancies: Implications for Immunotherapy. Nat Rev Clin Oncol (2021) 18(November):729–44. doi: 10.1038/s41571-021-00518-9 PMC11090136 34117475

[31] Rahimi Koshkaki H Minasi S Ugolini A Trevisi G Napoletano C Zizzari IG. Immunohistochemical Characterization of Immune Infiltrate in Tumor Microenvironment of Glioblastoma. J Pers Med (2020) 10(3):1–16. doi: 10.3390/jpm10030112 PMC7564919 32899203

[32] Teo W-Y Elghetany MT Shen J Man T-K Li X Chintagumpala M. Therapeutic Implications of CD1d Expression and Tumor-Infiltrating Macrophages in Pediatric Medulloblastomas. J Neurooncol (2014) 120(2):293–301. doi: 10.1007/s11060-014-1572-5 25115738

[33] Nam SJ Kim Y-H Park JE Ra Y Khang SK Cho YH. Tumor-Infiltrating Immune Cell Subpopulations and Programmed Death Ligand 1 (PD-L1) Expression Associated With Clinicopathological and Prognostic Parameters in Ependymoma. Cancer Immunol Immunother (2019) 68(2):305–18. doi: 10.1007/s00262-018-2278-x PMC11028367 30483834

[34] Murata D Mineharu Y Arakawa Y Liu B Tanji M Yamaguchi M. High Programmed Cell Death 1 Ligand–1 Expression: Association With CD8+ T-Cell Infiltration and Poor Prognosis in Human Medulloblastoma. J Neurosurg (2018) 128(March):710–6. doi: 10.3171/2016.11.JNS16991 28474991

[35] Vermeulen JF Van Hecke W Adriaansen EJM Jansen MK Bouma RG Villacorta Hidalgo J. Prognostic Relevance of Tumor-Infiltrating Lymphocytes and Immune Checkpoints in Pediatric Medulloblastoma. Oncoimmunology (2018) 7(3):e1398877. doi: 10.1080/2162402X.2017.1398877 29399402 PMC5790383

[36] Theruvath J Sotillo E Mount CW Graef CM Delaidelli A Heitzeneder S. Locoregionally Administered B7-H3-Targeted CAR T Cells for Treatment of Atypical Teratoid / Rhabdoid Tumors. Nat Med (2020) 26:712–9. doi: 10.1038/s41591-020-0821-8 PMC7992505 32341579

[37] Maachani UB Tosi U David J Mukherjee S Christopher S. B7 E H3 as a Prognostic Biomarker and Therapeutic Target in Pediatric Central Nervous System Tumors. Transl Oncol (2020) 13(2):365–71. doi: 10.1016/j.tranon.2019.11.006 PMC6938869 31887631

[38] Thompson EM Brown M Dobrikova E Ramaswamy V Taylor MD Mclendon R. Poliovirus Receptor ( CD155 ) Expression in Pediatric Brain Tumors Mediates Oncolysis of Medulloblastoma and Pleomorphic Xanthoastrocytoma. J Neuropathol Exp Neurol (2018) 77(8):696–702. doi: 10.1093/jnen/nly045 29878245 PMC6044395

[39] Widodo SS Hutchinson RA Fang Y Mangiola S Neeson PJ Darcy PK. Toward Precision Immunotherapy Using Multiplex Immunohistochemistry and in Silico Methods to Define the Tumor Immune Microenvironment. Cancer Immunol Immunother (2021) 70(7):1811–20. doi: 10.1007/s00262-020-02801-7 PMC10991574 33389014

[40] Marcelis L Antoranz A Delsupehe A-M Biesemans P Ferreiro JF Debackere K. In-Depth Characterization of the Tumor Microenvironment in Central Nervous System Lymphoma Reveals Implications for Immune-Checkpoint Therapy. Cancer Immunol Immunother (2020) 69(9):1751–66. doi: 10.1007/s00262-020-02575-y PMC11027603 32335702

[41] Bernstock JD Vicario N Rong L Valdes PA Choi BD Chen JA. A Novel *in Situ* Multiplex Immunofluorescence Panel for the Assessment of Tumor Immunopathology and Response to Virotherapy in Pediatric Glioblastoma Reveals a Role for Checkpoint Protein Inhibition. Oncoimmunology (2019) 8(12):1–12. doi: 10.1080/2162402X.2019.1678921 PMC6844311 31741780

[42] Lundberg E Borner GHH. Spatial Proteomics: A Powerful Discovery Tool for Cell Biology. Nat Rev Mol Cell Biol (2019) 20:285–302. doi: 10.1038/s41580-018-0094-y 30659282

[43] Taylor MJ Lukowski JK Anderton CR. Spatially Resolved Mass Spectrometry at the Single Cell: Recent Innovations in Proteomics and Metabolomics. J Am Soc Mass Spectrom (2021) 32:872–984. doi: 10.1021/jasms.0c00439 33656885 PMC8033567

[44] Black S Phillips D Hickey JW Kennedy-darling J Venkataraaman VG Samusik N. CODEX Multiplexed Tissue Imaging With DNA-Conjugated Antibodies. Nat Protoc (2021) 16:3802–35. doi: 10.1038/s41596-021-00556-8 PMC8647621 34215862

[45] Phillips D Schürch CM Khodadoust MS Kim YH Nolan GP Jiang S. Highly Multiplexed Phenotyping of Immunoregulatory Proteins in the Tumor Microenvironment by CODEX Tissue Imaging. Front Immunol (2021) 12(May):1–12. doi: 10.3389/fimmu.2021.687673 PMC8170307 34093591

[46] Jaye DL Bray RA Gebel HM Harris WAC Waller EK. Translational Applications of Flow Cytometry in Clinical Practice. J Immunol (2012) 188:4715–9. doi: 10.4049/jimmunol.1290017 22556132

[47] Volovitz I Shapira N Ezer H Gafni A Lustgarten M Alter T. A non-Aggressive, Highly Efficient, Enzymatic Method for Dissociation of Human Brain-Tumors and Brain-Tissues to Viable Single-Cells. BMC Neurosci (2016) 17(1):1–10. doi: 10.1186/s12868-016-0262-y 27251756 PMC4888249

[48] Leelatian N Doxie DB Greenplate AR Sinnaeve J Ihrie RA Irish JM. Preparing Viable Single Cells From Human Tissue and Tumors for Cytomic Analysis. Curr Protoc Mol Biol (2017) 118:1–31. doi: 10.1002/cpmb.37 28369679 PMC5518778

[49] Woroniecka K Chongsathidkiet P Elsamadicy A Farber H Cui X Fecci PE. Flow Cytometric Identification of Tumor-Infiltrating Lymphocytes From Glioblastoma. Methods Mol Biol (2018) 1741:221–6. doi: 10.1007/978-1-4939-7659-1_18 PMC6825407 29392704

[50] Cossarizza A Chang H Radbruch A Andr I Martin B Foster J. Guidelines for the Use of Flow Cytometry and Cell Sorting in Immunological Studies *. Eur J Immunol (2017) 47(October 2017):1584–797. doi: 10.1002/eji.201646632 PMC9165548 29023707

[51] Johnson S Nguyen V Coder D. Assessment of Cell Viability. Curr Protoc Cytom (2013) SUPPL.64):1–26. doi: 10.1002/0471142956.cy0902s64 23546778

[52] Bradford J Buller G. Dead Cell Stains in Flow Cytometry: A Comprehensive Analysis. Available at: http://www.invitrogen.com/etc/medialib/en/filelibrary/cell_tissue_analysis/pdfs.Par.45053.File.tmp/ISAC-2008-DEAD-CELL-STAINS-IN-FLOW-CYTOMETRY-A-COMPREHENSIVE-ANALYSISl.pdf.

[53] Hokland P Heron I. Lymphocyte Isolation Estimate of Total Leucocyte and Differential Counts Preparation of Ery Throcy Tes. J Imminulogical Methods (1980) 32:31–9. doi: 10.1016/0022-1759(80)90114-3

[54] Dagur PK McCoy JP. Collection, Storage, and Preparation of Human Blood Cells. Curr Protoc Cytom (2015) 2015:5.1.1–5.1.16. doi: 10.1002/0471142956.cy0501s73 PMC4524540 26132177

[55] Cossarizza A Cossarizza A Chang H Radbruch A Acs A Adam D. Guidelines for the Use of Flow Cytometry and Cell Sorting in Immunological Studies ( Second Edition ). Eur J Immunol (2019) 49:1457–973. doi: 10.1002/eji.201970107 PMC7350392 31633216

[56] Griesinger AM Birks DK Donson AM Amani V Hoffman LM Waziri A. Characterization of Distinct Immunophenotypes Across Pediatric Brain Tumor Types. J Immunol (2013) 191(9):1–19. doi: 10.4049/jimmunol.1301966 24078694 PMC3827919

[57] Klemm F Maas RR Bowman RL Kornete M Soukup K Nassiri S. Interrogation of the Microenvironmental Landscape in Brain Tumors Reveals Disease-Specific Alterations of Immune Cells. Cell (2020) 181(7):1643–1660.e17. doi: 10.1016/j.cell.2020.05.007 32470396 PMC8558904

[58] Riva G Nasillo V Ottomano AM Bergonzini G Paolini A Forghieri F. Multiparametric Flow Cytometry for MRD Monitoring in Hematologic Malignancies: Clinical Applications and New Challenges. Cancers (Basel) (2021) 13(18):4582. doi: 10.3390/cancers13184582 34572809 PMC8470441

[59] van der Velden VHJ Hochhaus A Cazzaniga G Szczepanski T Gabert J van Dongen JJM. Detection of Minimal Residual Disease in Hematologic Malignancies by Real-Time Quantitative PCR: Principles, Approaches, and Laboratory Aspects. Leukemia (2003) 17(6):1013–34. doi: 10.1038/sj.leu.2402922 12764363

[60] Chongsathidkiet P Jackson C Koyama S Loebel F Cui X Farber SH. Sequestration of T-Cells in Bone Marrow in the Setting of Glioblastoma and Other Intracranial Tumors. Nat Methods (2018) 24(9):1459–68. doi: 10.1038/s41591-018-0135-2 PMC6129206 30104766

[61] Alban TJ Alvarado AG Sorensen MD Bayik D Volovetz J Serbinowski E. Global Immune Fingerprinting in Glioblastoma Patient Peripheral Blood Reveals Immune-Suppression Signatures Associated With Prognosis. JCI Insight (2018) 3(21):1–15. doi: 10.1172/jci.insight.122264 PMC6238746 30385717

[62] Verhaak RGW Hoadley KA Purdom E Wang V Qi Y Wilkerson MD. An Integrated Genomic Analysis Identifies Clinically Relevant Subtypes of Glioblastoma Characterized by Abnormalities in PDGFRA, IDH1, EGFR and NF1. Cancer Cell (2010) 17(1):98. doi: 10.1016/j.ccr.2009.12.020 20129251 PMC2818769

[63] Byron S Van Keuren-Jensen K Engelthaler D Carpten JD Craig DW. Translating RNA Sequencing Into Clinical Diagnostics: Opportunities and Challenges. Nat Rev Immunol (2016) 17:257–71. doi: 10.1038/nrg.2016.10 PMC7097555 26996076

[64] van Belzen IAEM Cai C van Tuil M Badloe S Strengman E Janse A. Systematic Discovery of Gene Fusions in Pediatric Cancer by Integrating RNA-Seq and WGS. Genomics (2021) 2012:2021.08.31.458342. doi: 10.1101/2021.08.31.458342 PMC10318758 37400763

[65] Vanguilder HD Vrana KE Freeman WM Facility GC. Twenty-Five Years of Quantitative PCR for Gene Expression. Biotechniques (2008) 44(5):619–26. doi: 10.2144/000112776 18474036

[66] Rogawski DS Vitanza NA Gauthier AC Ramaswamy V Koschmann C. Integrating RNA Sequencing Into Neuro-Oncology Practice. Trans Res J Lab Clin Med (2017) 189:93–104. doi: 10.1016/j.trsl.2017.06.013 PMC5659901 28746860

[67] Mantione KJ Kream RM Kuzelova H Ptacek R Raboch J Samuel JM. Comparing Bioinformatic Gene Expression Profiling Methods: Microarray and RNA-Seq. Mol Biol (2014) 20:138–41. doi: 10.12659/MSMBR.892101 PMC4152252 25149683

[68] M’Boutchou MN van Kempen LC. Analysis of the Tumor Microenvironment Transcriptome *via* NanoString mRNA and miRNA Expression Profiling. Methods Mol Biol (2016) 1458:291–310. doi: 10.1007/978-1-4939-3801-8_21 27581030

[69] Veldman-jones MH Brant R Rooney C Geh C Emery H Harbron CG. Evaluating Robustness and Sensitivity of the NanoString Technologies Ncounter Platform to Enable Multiplexed Gene Expression Analysis of Clinical Samples. Cancer Res (2015) 75:2587–93. doi: 10.1158/0008-5472.CAN-15-0262 26069246

[70] Cesano A. Ncounter ® PanCancer Immune Profiling Panel (NanoString Technologies , Inc. J Immunother Cancer (2015) 3:42. doi: 10.1186/s40425-015-0088-7 26674611 PMC4678588

[71] Hong M Tao S Zhang L Diao L-T Huang X Huang S. RNA Sequencing: New Technologies and Applications in Cancer Research. J Hematol Oncol (2020) 13(1):166. doi: 10.1186/s13045-020-01005-x 33276803 PMC7716291

[72] Kuksin M Gautheret D. ScienceDirect Applications of Single-Cell and Bulk RNA Sequencing in. ScienceDirect (2021) 149:193–210. doi: 10.1016/j.ejca.2021.03.005 33866228

[73] Smith CC Bixby LM Miller KL Selitsky SR Bortone DS Hoadley KA. Using RNA Sequencing to Characterize the Tumor Microenvironment. Methods Mol Biol (2019) 2055:245–72. doi: 10.1007/978-1-4939-9773-2_12 31502156

[74] Jones DTW Kocialkowski S Liu L Pearson DM Ba LM Ichimura K. Tandem Duplication Producing a Novel Oncogenic BRAF Fusion Gene Defines the Majority of Pilocytic Astrocytomas. Cancer Immunol Immunother (2008) 68(21):8673–8. doi: 10.1158/0008-5472.CAN-08-2097 PMC2577184 18974108

[75] Parker M Mohankumar KM Punchihewa C Weinlich R Dalton JD Li Y. NF- K B Signalling in Ependymoma. Nature (2014) 506:451–5. doi: 10.1038/nature13109 PMC4050669 24553141

[76] Becht E Giraldo NA Lacroix L Buttard B Elarouci N Petitprez F. Estimating the Population Abundance of Tissue-Infiltrating Immune and Stromal Cell Populations Using Gene Expression. Genome Biol (2016) 17:218. doi: 10.1186/s13059-016-1070-5 27765066 PMC5073889

[77] Koch CM Chiu SF Akbarpour M Bharat A Ridge KM Bartom ET. A Beginner’s Guide to Analysis of RNA Sequencing Data. Am J Respir Cell Mol Biol (2018) 59(2):145–57. doi: 10.1165/rcmb.2017-0430TR PMC6096346 29624415

[78] Yang Y Sun H Zhang Y Zhang T Gong J Wei Y. Dimensionality Reduction by UMAP Reinforces Sample Heterogeneity Analysis in Bulk Transcriptomic Data. Cell Rep (2021) 36(4):109442. doi: 10.1016/j.celrep.2021.109442 34320340

[79] Bergthold G Bandopadhayay P Hoshida Y Ramkissoon S Ramkissoon L Rich B. Expression Profiles of 151 Pediatric Low-Grade Gliomas Reveal Molecular Differences Associated With Location and Histological Subtype. Neuro Oncol (2015) 17(11):1486–96. doi: 10.1093/neuonc/nov045 PMC4648300 25825052

[80] Zakrzewski K Jarząb M Pfeifer A Oczko-Wojciechowska M Jarząb B Liberski PP. Transcriptional Profiles of Pilocytic Astrocytoma are Related to Their Three Different Locations, But Not to Radiological Tumor Features. BMC Cancer (2015) 15(1):778. doi: 10.1186/s12885-015-1810-z 26497896 PMC4619381

[81] Northcott PA Buchhalter I Morrissy AS Hovestadt V Weischenfeldt J Ehrenberger T. The Whole-Genome Landscape of Medulloblastoma Subtypes. Nature (2017) 547(7663):311–7. doi: 10.1038/nature22973 PMC5905700 28726821

[82] Wang L-B Karpova A Gritsenko MA Kyle JE Cao S Li Y. Proteogenomic and Metabolomic Characterization of Human Glioblastoma. Cancer Cell (2021) 39(4):509–528.e20. doi: 10.1016/j.ccell.2021.01.006 33577785 PMC8044053

[83] Donson AM Birks DK Schittone SA Kleinschmidt-DeMasters BK Sun DY Hemenway MF. Increased Immune Gene Expression and Immune Cell Infiltration in High Grade Astrocytoma Distinguish Long From Short-Term Survivors. J Immunol (2012) 189(4):1920–7. doi: 10.4049/jimmunol.1103373 PMC3411857 22802421

[84] Love MI Huber W Anders S. Moderated Estimation of Fold Change and Dispersion for RNA-Seq Data With Deseq2. Genome Biol (2014) 15(550):1–21. doi: 10.1186/s13059-014-0550-8 PMC4302049 25516281

[85] Robinson MD Mccarthy DJ Smyth GK. Edger: A Bioconductor Package for Differential Expression Analysis of Digital Gene Expression Data. Bioinformatics (2010) 26(1):139–40. doi: 10.1093/bioinformatics/btp616 PMC2796818 19910308

[86] Yin W Tang G Zhou Q Cao Y Li H Fu X. Expression Profile Analysis Identifies a Novel Five-Gene Signature to Improve Prognosis Prediction of Glioblastoma. Front Genet (2019) 10:419. doi: 10.3389/fgene.2019.00419 31130992 PMC6509566

[87] Subramanian A Tamayo P Mootha VK Mukherjee S Ebert BL. Gene Set Enrichment Analysis: A Knowledge-Based Approach for Interpreting Genome-Wide. PNAS (2005) 102(43):15545–50. doi: 10.1073/pnas.0506580102 PMC1239896 16199517

[88] Kanehisa M Goto S. KEGG: Kyoto Encyclopedia of Genes and Genomes. Nucleic Acids Res (2000) 28(1):27–30. doi: 10.1093/nar/28.1.27 10592173 PMC102409

[89] Liberzon A Subramanian A Pinchback R Thorvaldsdóttir H Tamayo P Mesirov JP. Molecular Signatures Database (MSigDB) 3.0. Bioinformatics (2011) 27(12):1739–40. doi: 10.1093/bioinformatics/btr260 PMC3106198 21546393

[90] Mackay A Burford A Molinari V Jaspan T Varlet P Jones C. Molecular, Pathological, Radiological, and Immune Profiling of Non-Brainstem Pediatric High-Grade Glioma From the HERBY Phase II Randomized Trial. Cancer Cell (2018) 33:829–42. doi: 10.1016/j.ccell.2018.04.004 PMC5956280 29763623

[91] Bockmayr M Mohme M Klauschen F Winkler B Budczies J Rutkowski S. Subgroup-Specific Immune and Stromal Microenvironment in Medulloblastoma. Oncoimmunology (2018) 7(9):e1462430. doi: 10.1080/2162402X.2018.1462430 30228931 PMC6140816

[92] Lin GL Nagaraja S Filbin MG Suvà ML Vogel H Monje M. Non-Inflammatory Tumor Microenvironment of Diffuse Intrinsic Pontine Glioma. Acta Neuropathol Commun (2018) 6(51):1–12. doi: 10.1186/s40478-018-0553-x 29954445 PMC6022714

[93] van der Sijde F Li Y Schraauwen R de Koning W van Eijck C Mustafa D. RNA From Stabilized Whole Blood Enables More Comprehensive Immune Gene Expression Profiling Compared to RNA From Peripheral Blood Mononuclear Cells. PloS One (2020) 15(6):1–12. doi: 10.1371/journal.pone.0235413 PMC7319339 32589655

[94] Turner JD Williamson R Almefty KK Nakaji P Porter R Tse V. The Many Roles of microRNAs in Brain Tumor Biology. Neurosurg Focus (2010) 28(1):E3. doi: 10.3171/2009.10.FOCUS09207 20043718

[95] Yeh M Wang YY Yoo JY Oh C Otani Y Kang JM. MicroRNA − 138 Suppresses Glioblastoma Proliferation Through Downregulation of CD44. Sci Rep (2021) 11:9219. doi: 10.1038/s41598-021-88615-8 33911148 PMC8080729

[96] Aloizou A Pateraki G Siokas V Mentis AA Liampas I Lazopoulos G. The Role of MiRNA-21 in Gliomas: Hope for a Novel Therapeutic Intervention? Toxicol Rep (2020) 7(November):1514–30. doi: 10.1016/j.toxrep.2020.11.001 PMC7677650 33251119

[97] Blass E Ott PA. Advances in the Development of Personalized Neoantigen- ​ Based Therapeutic Cancer Vaccines. Nat Rev Clin Oncol (2021) 18:215–29. doi: 10.1038/s41571-020-00460-2 PMC7816749 33473220

[98] Zhang Z Lu M Qin Y Gao W Tao L Su W. Neoantigen: A New Breakthrough in Tumor Immunotherapy. Front Immunol (2021) 12(April):1–9. doi: 10.3389/fimmu.2021.672356 PMC8085349 33936118

[99] Vogelstein B Papadopoulos N Velculescu VE Zhou S Diaz LA Kinzler KW. CancerGenomeLandscape. Sci (80- ). (2013) 339(6127):1546–58. doi: 10.1126/science.1235122 PMC3749880 23539594

[100] Blaeschke F Paul MC Schuhmann MU Rabsteyn A Schroeder C Casadei N. Low Mutational Load in Pediatric Medulloblastoma Still Translates Into Neoantigens as Targets for Specific T-Cell Immunotherapy. Cytotherapy (2019) 21(9):973–86. doi: 10.1016/j.jcyt.2019.06.009 31351799

[101] Rivero-hinojosa S Grant M Panigrahi A Zhang H Caisova V Bollard CM. Proteogenomic Discovery of Neoantigens Facilitates Personalized Multi-Antigen Targeted T Cell Immunotherapy for Brain Tumors. Nat Commun (2021) 12(6689):1–15. doi: 10.1038/s41467-021-26936-y 34795224 PMC8602676

[102] Nejo T Matsushita H Karasaki T Nomura M Saito K Tanaka S. Reduced Neoantigen Expression Revealed by Longitudinal Multiomics as a Possible Immune Evasion Mechanism in Glioma. Cancer Immunol Res (2019) 7(7):1148–62. doi: 10.1158/2326-6066.CIR-18-0599 31088845

[103] Keskin DB Anandappa AJ Sun J Tirosh I Mathewson ND Li S. Neoantigen Vaccine Generates Intratumoral T Cell Responses in Phase Ib Glioblastoma Trial. Nature (2018) 565:234–9. doi: 10.1038/s41586-018-0792-9 PMC6546179 30568305

[104] Hilf N Kuttruff-Coqui S Frenzel K Bukur V Stevanović S Gouttefangeas C. Actively Personalized Vaccination Trial for Newly Diagnosed Glioblastoma. Nature (2019) 565(7738):240–5. doi: 10.1038/s41586-018-0810-y 30568303

[105] Filbin MG Tirosh I Hovestadt V Shaw ML Escalante LE Mathewson ND. Developmental and Oncogenic Programs in H3K27M Gliomas Dissected by Single-Cell RNA-Seq. Sci (80- ) (2018) 360(6386):331–5. doi: 10.1126/science.aao4750 PMC5949869 29674595

[106] Neftel C Laffy J Filbin MG Hara T Shore ME Rahme GJ. An Integrative Model of Cellular States, Plasticity, and Genetics for Glioblastoma. Cell (2019) 178(4):835–849.e21. doi: 10.1016/j.cell.2019.06.024 31327527 PMC6703186

[107] Schafflick D Xu CA Hartlehnert M Cole M Schulte-Mecklenbeck A Lautwein T. Integrated Single Cell Analysis of Blood and Cerebrospinal Fluid Leukocytes in Multiple Sclerosis. Nat Commun (2020) 11(1):247. doi: 10.1038/s41467-019-14118-w 31937773 PMC6959356

[108] Rubio-Perez C Planas-Rigol E Trincado JL Bonfill-Teixidor E Arias A Marchese D. Immune Cell Profiling of the Cerebrospinal Fluid Enables the Characterization of the Brain Metastasis Microenvironment. Nat Commun (2021) 12:1503. doi: 10.1038/s41467-021-21789-x 33686071 PMC7940606

[109] Slyper M Porter CBM Ashenberg O Waldman J Drokhlyansky E Wakiro I. A Single-Cell and Single-Nucleus RNA-Seq Toolbox for Fresh and Frozen Human Tumors. Nat Med (2020) 26(5):792–802. doi: 10.1038/s41591-020-0844-1 32405060 PMC7220853

[110] Picelli S Björklund ÅK Faridani OR Sagasser S Winberg G Sandberg R. Smart-Seq2 for Sensitive Full-Length Transcriptome Profiling in Single Cells. Nat Methods (2013) 10(11):1096–8. doi: 10.1038/nmeth.2639 24056875

[111] Hayashi T Ozaki H Sasagawa Y Umeda M Danno H Nikaido I. Single-Cell Full-Length Total RNA Sequencing Uncovers Dynamics of Recursive Splicing and Enhancer RNAs. Nat Commun (2018) 9(1):619. doi: 10.1038/s41467-018-02866-0 29434199 PMC5809388

[112] Sheng K Cao W Niu Y Deng Q Zong C. Effective Detection of Variation in Single-Cell Transcriptomes Using MATQ-Seq. Nat Methods (2017) 14(3):267–70. doi: 10.1038/nmeth.4145 28092691

[113] Macosko EZ Basu A Satija R Nemesh J Shekhar K Goldman M. Highly Parallel Genome-Wide Expression Profiling of Individual Cells Using Nanoliter Droplets. Cell (2015) 161(5):1202–14. doi: 10.1016/j.cell.2015.05.002 PMC4481139 26000488

[114] Klein AM Mazutis L Akartuna I Tallapragada N Veres A Li V. Droplet Barcoding for Single Cell Transcriptomics Applied to Embryonic Stem Cells. Cell (2015) 161(5):1187–201. doi: 10.1016/j.cell.2015.04.044 PMC4441768 26000487

[115] Satija R Farrell JA Gennert D Schier AF Regev A. Spatial Reconstruction of Single-Cell Gene Expression Data. Nat Biotechnol (2015) 33(5):495–502. doi: 10.1038/nbt.3192 25867923 PMC4430369

[116] Wolf FA Angerer P Theis FJ. SCANPY: Large-Scale Single-Cell Gene Expression Data Analysis. Genome Biol (2018) 19(1):15. doi: 10.1186/s13059-017-1382-0 29409532 PMC5802054

[117] Gojo J Englinger B Jiang L Hübner JM Shaw ML Hack OA. Single-Cell RNA-Seq Reveals Cellular Hierarchies and Impaired Developmental Trajectories in Pediatric Ependymoma. Cancer Cell (2020) 38(1):44–59.e9. doi: 10.1016/j.ccell.2020.06.004 32663469 PMC7479515

[118] Hovestadt V Smith KS Bihannic L Filbin MG Shaw ML Baumgartner A. Resolving Medulloblastoma Cellular Architecture by Single-Cell Genomics. Nature (2019) 572(7767):74–9. doi: 10.1038/s41586-019-1434-6 PMC6754173 31341285

[119] Jessa S Blanchet-Cohen A Krug B Vladoiu M Coutelier M Faury D. Stalled Developmental Programs at the Root of Pediatric Brain Tumors. Nat Genet (2019) 51(12):1702–13. doi: 10.1038/s41588-019-0531-7 PMC6885128 31768071

[120] Vladoiu MC El-Hamamy I Donovan LK Farooq H Holgado BL Sundaravadanam Y. Childhood Cerebellar Tumours Mirror Conserved Fetal Transcriptional Programs. Nature (2019) 572(7767):67–73. doi: 10.1038/s41586-019-1158-7 31043743 PMC6675628

[121] Alghamri MS McClellan BL Avvari RP Thalla R Carney S Hartlage MS. G-CSF Secreted by Mutant IDH1 Glioma Stem Cells Abolishes Myeloid Cell Immunosuppression and Enhances the Efficacy of Immunotherapy. Sci Adv (2021) 7(40):eabh3243. doi: 10.1126/sciadv.abh3243 34586841 PMC8480930

[122] Sankowski R Böttcher C Masuda T Geirsdottir L Sagar Sindram E. Mapping Microglia States in the Human Brain Through the Integration of High-Dimensional Techniques. Nat Neurosci (2019) 22(12):2098–110. doi: 10.1038/s41593-019-0532-y 31740814

[123] Pombo Antunes AR Scheyltjens I Lodi F Messiaen J Antoranz A Duerinck J. Single-Cell Profiling of Myeloid Cells in Glioblastoma Across Species and Disease Stage Reveals Macrophage Competition and Specialization. Nat Neurosci (2021) 24(4):595–610. doi: 10.1038/s41593-020-00789-y 33782623

[124] Ochocka N Segit P Walentynowicz KA Wojnicki K Cyranowski S Swatler J. Single-Cell RNA Sequencing Reveals Functional Heterogeneity of Glioma-Associated Brain Macrophages. Nat Commun (2021) 12(1):1151. doi: 10.1038/s41467-021-21407-w 33608526 PMC7895824

[125] Chen AX Gartrell RD Zhao J Upadhyayula PS Zhao W Yuan J. Single-Cell Characterization of Macrophages in Glioblastoma Reveals MARCO as a Mesenchymal Pro-Tumor Marker. Genome Med (2021) 13(1):88. doi: 10.1186/s13073-021-00906-x 34011400 PMC8136167

[126] Reitman ZJ Paolella BR Bergthold G Pelton K Becker S Jones R. Mitogenic and Progenitor Gene Programmes in Single Pilocytic Astrocytoma Cells. Nat Commun (2019) 10:3731. doi: 10.1038/s41467-019-11493-2 31427603 PMC6700116

[127] Pai JA Satpathy AT. High-Throughput and Single-Cell T Cell Receptor Sequencing Technologies. Nat Methods (2021) 18(8):881–92. doi: 10.1038/s41592-021-01201-8 PMC9345561 34282327

[128] Redmond D Poran A Elemento O. Single-Cell TCRseq: Paired Recovery of Entire T-Cell Alpha and Beta Chain Transcripts in T-Cell Receptors From Single-Cell RNAseq. Genome Med (2016) 8(1):80. doi: 10.1186/s13073-016-0335-7 27460926 PMC4962388

[129] Stubbington MJT Lönnberg T Proserpio V Clare S Speak AO Dougan G. T Cell Fate and Clonality Inference From Single-Cell Transcriptomes. Nat Methods (2016) 13(4):329–32. doi: 10.1038/nmeth.3800 PMC4835021 26950746

[130] Rizzetto S Koppstein DNP Samir J Singh M Reed JH Cai CH. B-Cell Receptor Reconstruction From Single-Cell RNA-Seq With VDJPuzzle. Bioinformatics (2018) 34(16):2846–7. doi: 10.1093/bioinformatics/bty203 29659703

[131] Mathewson ND Ashenberg O Tirosh I Gritsch S Perez EM Marx S. Inhibitory CD161 Receptor Identified in Glioma-Infiltrating T Cells by Single-Cell Analysis. Cell Anal (2021) 184(5):1281–98. doi: 10.1016/j.cell.2021.01.022 PMC7935772 33592174

[132] Ruiz-Moreno C Keramati F Brazda P Megchelenbrink W te PB Boshuisen K. Reprogramming of Pro-Tumor Macrophages by Hydroxychloroquine in an Abdominally Metastasized Diffuse Midline Glioma. Oncology (2021) 2021:2021.07.19.21259735. doi: 10.1101/2021.07.19.21259735

[133] Prakadan SM Alvarez-Breckenridge CA Markson SC Kim AE Klein RH Nayyar N. Genomic and Transcriptomic Correlates of Immunotherapy Response Within the Tumor Microenvironment of Leptomeningeal Metastases. Nat Commun (2021) 12(1):5955. doi: 10.1038/s41467-021-25860-5 34642316 PMC8511044

[134] Newman AM Steen CB Liu CL Gentles AJ Chaudhuri AA Scherer F. Determining Cell Type Abundance and Expression From Bulk Tissues With Digital Cytometry. Nat Biotechnol (2019) 37(7):773–82. doi: 10.1038/s41587-019-0114-2 PMC6610714 31061481

[135] Wang X Park J Susztak K Zhang NR Li M. Bulk Tissue Cell Type Deconvolution With Multi-Subject Single-Cell Expression Reference. Nat Commun (2019) 10(1):380. doi: 10.1038/s41467-018-08023-x 30670690 PMC6342984

[136] Tsoucas D Dong R Chen H Zhu Q Guo G Yuan G-C. Accurate Estimation of Cell-Type Composition From Gene Expression Data. Nat Commun (2019) 10(1):2975. doi: 10.1038/s41467-019-10802-z 31278265 PMC6611906

[137] Qi Z Liu Y Mints M Mullins R Sample R Law T. Single-Cell Deconvolution of Head and Neck Squamous Cell Carcinoma. Cancers (Basel) (2021) 13(6):1230. doi: 10.3390/cancers13061230 33799782 PMC7999850

[138] Armingol E Officer A Harismendy O Lewis NE. Deciphering Cell–Cell Interactions and Communication From Gene Expression. Nat Rev Genet (2021) 22(2):71–88. doi: 10.1038/s41576-020-00292-x 33168968 PMC7649713

[139] Stoeckius M Hafemeister C Stephenson W Houck-Loomis B Chattopadhyay PK Swerdlow H. Simultaneous Epitope and Transcriptome Measurement in Single Cells. Nat Methods (2017) 14(9):865–8. doi: 10.1038/nmeth.4380 PMC5669064 28759029

[140] Ståhl PL Salmén F Vickovic S Lundmark A Navarro JF Magnusson J. Visualization and Analysis of Gene Expression in Tissue Sections by Spatial Transcriptomics. Science (2016) 353(6294):78–82. doi: 10.1126/science.aaf2403 27365449

[141] Rodriques SG Stickels RR Goeva A Martin CA Murray E Vanderburg CR. Slide-Seq: A Scalable Technology for Measuring Genome-Wide Expression at High Spatial Resolution. Sci (80- ). (2019) 363(6434):1463–7. doi: 10.1126/science.aaw1219 PMC6927209 30923225

[142] Stickels RR Murray E Kumar P Li J Marshall JL Di Bella DJ. Highly Sensitive Spatial Transcriptomics at Near-Cellular Resolution With Slide-Seqv2. Nat Biotechnol (2021) 39(3):313–9. doi: 10.1038/s41587-020-0739-1 PMC8606189 33288904

[143] Ravi VM Neidert N Will P Joseph K Maier JP Kückelhaus J. T-Cell Dysfunction in the Glioblastoma Microenvironment is Mediated by Myeloid Cells Releasing Interleukin-10. Nat Commun (2022) 13(1):925. doi: 10.1038/s41467-022-28523-1 35177622 PMC8854421

[144] Hunter MV Moncada R Weiss JM Yanai I White RM. Spatially Resolved Transcriptomics Reveals the Architecture of the Tumor-Microenvironment Interface. Nat Commun (2021) 12(1):6278. doi: 10.1038/s41467-021-26614-z 34725363 PMC8560802

[145] Poch M Hall M Joerger A Kodumudi K Beatty M Innamarato PP. Expansion of Tumor Infiltrating Lymphocytes (TIL) From Bladder Cancer. Oncoimmunology (2018) 7(9):1–7. doi: 10.1080/2162402X.2018.1476816 PMC6140546 30228944

[146] Besser MJ Shapira-Frommer R Treves AJ Zippel D Itzhaki O Hershkovitz L. Clinical Responses in a Phase II Study Using Adoptive Transfer of Short-Term Cultured Tumor Infiltration Lymphocytes in Metastatic Melanoma Patients. Clin Cancer Res (2010) 16(9):2646–55. doi: 10.1158/1078-0432.CCR-10-0041 20406835

[147] Stevanović S Draper LM Langhan MM Campbell TE Kwong ML Wunderlich JR. Complete Regression of Metastatic Cervical Cancer After Treatment With Human Papillomavirus-Targeted Tumor-Infiltrating T Cells. J Clin Oncol (2015) 33(14):1543–50. doi: 10.1200/JCO.2014.58.9093 PMC4417725 25823737

[148] Fujita K Ikarashi H Akiteru K. Prolonged Disease-Free Period in Patients With Advanced Epithelial Ovarian Cancer After Adoptive Transfer of Tumor-Infiltrating Lymphocytes. Clin Cancer Res (1995) 1(May):501–7. 9816009

[149] Brinke AT Marek-Trzonkowska N Mansilla MJ Turksma AW Piekarska K Iwaszkiewicz-Grzes D. Monitoring T-Cell Responses in Translational Studies: Optimization of Dye-Based Proliferation Assay for Evaluation of Antigen-Specific Responses. Front Immunol (2017) 8(DEC):1–15. doi: 10.3389/fimmu.2017.01870 29312346 PMC5742609

[150] Riddell SR Greenberg PD. The Use of Anti-CD3 and Anti-CD28 Monoclonal Antibodies to Clone and Expand Human Antigen-Specific T Cells. J Immunol Methods (1990) 128(2):189–201. doi: 10.1016/0022-1759(90)90210-M 1691237

[151] Hulen TM Chamberlain CA Svane IM Met Ö. ACT Up TIL Now: The Evolution of Tumor-Infiltrating Lymphocytes in Adoptive Cell Therapy for the Treatment of Solid Tumors. Immuno (2021) 1(3):194–211. doi: 10.3390/immuno1030012

[152] Alessandro Moretta B Pantaleo G Moreti L Cerottini J Maria Cristina Mingari A. Clonal Analysis of HLA-DR Expression and Cytolytic Activity*. J Exp Med (1983) 157(February):743–54. doi: 10.1084/jem.157.2.743 PMC2186923 6600491

[153] Van Den Berg JH Heemskerk B Van Rooij N Gomez-Eerland R Michels S Van Zon M. Tumor Infiltrating Lymphocytes (TIL) Therapy in Metastatic Melanoma: Boosting of Neoantigen-Specific T Cell Reactivity and Long-Term Follow-Up. J Immunother Cancer (2020) 8(2):1–11. doi: 10.1136/jitc-2020-000848 PMC7406109 32753545

[154] Hong JJ Rosenberg SA Dudley ME Yang JC Whit DE Butman JA. Successful Treatment of Melanoma Brain Metastases With Adoptive Cell Therapy. Clin Cancer Res (2010) 16(19):4892–8. doi: 10.1158/1078-0432.CCR-10-1507 PMC6291850 20719934

[155] Liu Z Meng Q Bartek J Poiret T Persson O Rane L. Tumor-Infiltrating Lymphocytes (TILs) From Patients With Glioma. Oncoimmunology (2017) 6(2):e1252894. doi: 10.1080/2162402X.2016.1252894 28344863 PMC5353900

[156] Li Y Polyak D Lamsam L Connolly ID Johnson E Khoeur LK. Comprehensive RNA Analysis of CSF Reveals a Role for CEACAM6 in Lung Cancer Leptomeningeal Metastases. NPJ Precis Oncol (2021) 90:1–8. doi: 10.1038/s41698-021-00228-6 PMC8501028 34625644

[157] Chen P Roh W Reuben A Cooper ZA Spencer CN Prieto PA. Analysis of Immune Signatures in Longitudinal Tumor Samples Yields Insight Into Biomarkers of Response and Mechanisms of Resistance to Immune Checkpoint Blockade. Cancer Discovery (2016) 6:827–3. doi: 10.1158/2159-8290.CD-15-1545 PMC5082984 27301722

[158] Kretzschmar H. Brain Banking: Opportunities, Challenges and Meaning for the Future. Nat Rev Neurosci (2009) 10(1):70–8. doi: 10.1038/nrn2535 19050713

[159] Abbott A. Tissue-Bank Shortage: Brain Child. Nature (2011) 478(7370):442–3. doi: 10.1038/478442a 22031417

[160] Day BW Brett SW Wilson J Jeffree RL Jamieson PR Ensbey KS. Glioma Surgical Aspirate: A Viable Source of Tumor Tissue for Experimental Research. Cancers (Basel) (2013) 5(2):357–71. doi: 10.3390/cancers5020357 PMC3730332 24216981

[161] Vaskova M Tichy M Zamecnik J Liby P Kuzilkova D Vicha A. Cytometric Analysis of Cell Suspension Generated by Cavitron Ultrasonic Surgical Aspirator in Pediatric Brain Tumors. J Neurooncol (2019) 143(1):15–25. doi: 10.1007/s11060-019-03135-w 30827009

[162] Rao S Vazhayil V Nandeesh B Beniwal M Rao K Yasha T. Diagnostic Utility of CUSA Specimen in Histopathological Evaluation of Tumors of Central Nervous System. Neurol India (2020) 68(6):1385–8. doi: 10.4103/0028-3886.304072 33342873

[163] Jacobs JFM Idema AJ Bol KF Nierkens S Grauer OM Wesseling P. Regulatory T Cells and the PD-L1/PD-1 Pathway Mediate Immune Suppression in Malignant Human Brain Tumors. Neuro Oncol (2009) 11(4):394–402. doi: 10.1215/15228517-2008-104 19028999 PMC2743219

[164] Spitzer A Gritsch S Weisman HR Gonzalez Castro LN Nomura M Druck N. Mutant IDH Inhibitors Induce Lineage Differentiation in IDH-Mutant Oligodendroglioma. Oncology (2021) 2021:2021.11.16.21266364. doi: 10.1101/2021.11.16.21266364 PMC11096020 38579724

[165] Liu APY Smith KS Kumar R Paul L Bihannic L Lin T. Serial Assessment of Measurable Residual Disease in Medulloblastoma Liquid Biopsies. Cancer Cell (2021) 39(11):1519–1530.e4. doi: 10.1016/j.ccell.2021.09.012 34678152 PMC9620970

[166] Harry GJ. Microglia During Development and Aging. Pharmacol Ther (2013) 139(3):313–26. doi: 10.1016/j.pharmthera.2013.04.013 PMC3737416 23644076

[167] Gutmann DH Kettenmann H. Microglia/brain Macrophages as Central Drivers of Brain Tumor Pathobiology. Neuron (2019) 104(3):442–9. doi: 10.1016/j.neuron.2019.08.028 PMC7288606 31697921

[168] Sayour EJ McLendon P McLendon R De Leon G Reynolds R Kresak J. Increased Proportion of FoxP3+ Regulatory T Cells in Tumor Infiltrating Lymphocytes is Associated With Tumor Recurrence and Reduced Survival in Patients With Glioblastoma. Cancer Immunol Immunother (2015) 64(4):419–27. doi: 10.1007/s00262-014-1651-7 PMC4774199 25555571

[169] Patterson JD Henson JC Breese RO Bielamowicz KJ Rodriguez A. CAR T Cell Therapy for Pediatric Brain Tumors. Front Oncol (2020) 10:1582. doi: 10.3389/fonc.2020.01582 32903405 PMC7435009

